# The effectiveness and cost-effectiveness of population-level policies to reduce alcohol use: A systematic umbrella review

**DOI:** 10.17269/s41997-025-01013-9

**Published:** 2025-04-03

**Authors:** G. Emmanuel Guindon, Clement Li, Riya Trivedi, Umaima Abbas, Grace Xiong, Alisha Atri

**Affiliations:** 1 Centre for Health Economics and Policy Analysis, McMaster University, Hamilton, ON Canada; 2 Department of Health Research Methods, Evidence, and Impact, McMaster University, Hamilton, ON Canada; 3 Department of Economics, McMaster University, Hamilton, ON Canada; 4 Michael G. DeGroote School of Medicine, McMaster University, Hamilton, ON Canada; 5 Schulich School of Medicine & Dentistry, Western University, Windsor, ON Canada; 6 Department of Family Medicine, McMaster University, Hamilton, ON Canada

**Keywords:** Alcohol drinking, Cost-effectiveness analysis, Marketing, Public policy, Systematic review, Consommation d’alcool, Analyse coût-efficacité, Marketing, Politique publique, Revue systématique

## Abstract

**Objective:**

To systematically review and synthesize evidence from reviews about the effectiveness and cost-effectiveness of population-level policies to reduce alcohol use.

**Methods:**

We searched peer-reviewed literature using eight electronic bibliographic databases, grey literature using two databases, two search engines, and two working paper repositories, and examined references of included studies. At least two reviewers independently screened articles for inclusion, extracted detailed characteristics, and assessed the risk of bias of each included study. We considered all reviews that included studies which quantitatively examined the relationship between alcohol consumption and population-level policies that seek to regulate the public availability and marketing of alcoholic beverages. After screening according to a set of predetermined criteria, we included 32 reviews.

**Synthesis:**

We found consistent evidence that addressing alcohol availability (introducing or increasing minimum purchasing age, restrictions on temporal availability, decreasing outlet density, government monopolization) was associated with lower alcohol use; and a general lack of evidence on the associations between alcohol marketing (marketing self-regulation, advertising from government authorities, regulating the volume of advertising from alcohol manufacturers, and introducing warning labels) and alcohol consumption, which precludes any conclusions about these regulations. Additionally, we found scarce evidence about the cost-effectiveness of population-level policies to reduce alcohol use, which is likely due to the relatively low cost of implementation and enforcement of these policies.

**Conclusion:**

The Government of Ontario began expanding privatized alcohol sales in 2015 with further expansions starting in August 2024. Evidence from reviews suggests that this increase in availability will result in increased alcohol consumption.

**Supplementary Information:**

The online version contains supplementary material available at 10.17269/s41997-025-01013-9.

## Résumé

**Objectif:**

Notre objectif est d’examiner et synthétiser systématiquement les données probantes issues de revues sur l’efficacité et le rapport coût-efficacité des politiques de réduction de la consommation d’alcool au niveau de la population.

**Méthodes:**

Nous avons effectué des recherches de la littérature évaluée par des pairs à l’aide de huit bases de données bibliographiques électroniques, de la littérature grise à l’aide de deux bases de données, de deux moteurs de recherche et de deux référentiels de documents de travail, et avons examiné les références des études incluses. Au moins deux évaluateurs ont indépendamment examiné les articles à inclure, extrait des caractéristiques détaillées et évalué le risque de biais de chaque étude incluse. Nous avons pris en compte toutes les revues incluant des études examinant quantitativement la relation entre la consommation d’alcool et les politiques au niveau de la population visant à réglementer la disponibilité et le marketing des boissons alcoolisées. Nous avons inclus 32 revues.

**Synthèse:**

Nous avons trouvé des preuves cohérentes selon lesquelles la réduction de la disponibilité de l’alcool (introduction ou augmentation de l’âge minimum d’achat, restrictions sur la disponibilité temporelle, diminution de la densité des points de vente, monopolisation gouvernementale) était associée à une consommation d’alcool plus faible; et un manque général de preuves sur les associations entre le marketing de l’alcool (autorégulation du marketing, publicité des autorités gouvernementales, réglementation du volume de publicité des fabricants d’alcool et introduction d’étiquettes d’avertissement) et la consommation d’alcool, ce qui exclut toute conclusion sur ces réglementations. De plus, nous avons trouvé peu de preuves sur le rapport coût-efficacité des politiques visant à réduire la consommation d’alcool à l’échelle de la population, ce qui est probablement dû au coût relativement faible de mise en œuvre et d’application de ces politiques.

**Conclusion:**

Le gouvernement de l’Ontario a commencé à accroître la libéralisation de la vente d’alcool en 2015, avec une accélération de l’expansion des ventes d’alcool débutant en août 2024. Les données probantes provenant de revues indiquent que cette augmentation de la disponibilité entraînera une augmentation de la consommation d’alcool.

## Introduction

Alcohol consumption is currently the world’s third-largest risk factor for disease, attributable for about 5% of the global burden of disease, with clear links to conditions such as neuropsychiatric disorders, gastrointestinal diseases, cancer, intentional and unintentional injuries, cardiovascular diseases, and diabetes (Shield et al., 2020; World Health Organization, 2024). Moreover, several cancers (e.g., lip and oral cavity, pharynx, esophagus, colon and rectum, liver, breast, and larynx) are causally linked to alcohol use (Rehm et al., 2020). In youth, heavy episodic drinking, such as binge drinking, has been associated with long-term consequences including cognitive, structural, and functional brain changes and liver disease (Meister et al., 2018). Lasting developmental delays and intellectual disabilities can also arise from fetal alcohol exposure (World Health Organization, 2024).

Canadians are among the largest consumers of alcohol in the world. Recent cross-country data indicated that Canadians ≥ 15 years of age consumed annually 9.9 L of alcohol per capita as compared to the global average of 5.5 L per capita (World Health Organization, 2023). In addition, almost one fifth of Canadians over the age of 12 were classified as heavy drinkers in 2018 and 2019 (Statistics Canada, 2025). To make matters worse, there were substantial increases in alcohol sales during the COVID-19 pandemic (MacKillop et al., 2021; Zajacova et al., 2020; Zipursky et al., 2021). In 2020, the overall costs of alcohol use in Canada were estimated to be $19.7 billion (Canadian Substance Use Costs & Harms Scientific Working Group, 2023).

Given the clear risks associated with alcohol use and the substantive levels of alcohol consumption in Canada, federal, provincial, territorial, and municipal governments have an important role in developing and implementing evidence-informed policies to reduce alcohol consumption. These policies may include decreasing the accessibility and availability of alcohol (e.g., via taxation, minimum pricing, reducing outlet density, implementing a minimum age for sale, limiting hours of sale), and regulating the marketing and advertising of alcohol (e.g., implementing plain packaging and decreasing the volume of advertising).

A 2022 assessment of federal alcohol policies in Canada found that few evidence-based policies had been implemented by the federal government (Farkouh et al., 2024). More recently, Ontario’s Premier Doug Ford announced a major expansion of the sale of alcohol, despite calls to the Government of Ontario to develop and implement a comprehensive alcohol strategy (Government of Ontario, 2024; Spadorcia et al., 2024).

Our objective was to systematically review and synthesize evidence about the effectiveness and cost-effectiveness of population-level policies to reduce alcohol use. We drew upon literature from several disciplines including health economics, health policy, political science, and psychology to present comprehensive findings. We built on a rapid synthesis conducted in collaboration with the Canadian Partnership Against Cancer that examined the effectiveness and cost-effectiveness of population-level policies for reducing alcohol consumption (Guindon et al., 2021). The high number of reviews identified in the rapid synthesis called for a systematic umbrella review. Umbrella reviews (i.e., overview of existing reviews or review of reviews) are “ideal in highlighting if the evidence base around a topic or question is consistent or if contradictory or discrepant findings exist, and in exploring and detailing the reasons why. Investigation of the evidence with an umbrella review allows assessment and consideration of whether reviewers addressing similar review questions independently observe similar results and arrive at generally similar conclusions” (Aromataris et al., 2020, p. 362).

## Methods

A review protocol was prepared in advance and registered with PROSPERO (an international database of prospectively registered systematic reviews): protocol registration number CRD42022330386. No revisions were made to the protocol.

### Searches

We searched eight electronic bibliographic databases (MEDLINE, EconLit, Cochrane Library, Health Evidence, Health Systems Evidence, PubMed, PsychInfo, and Social Systems Evidence). Unpublished and gray literature was searched via the New York Academy of Medicine Grey Literature Report, Open Grey, Google, Google Scholar, and two working paper repositories (RePEc, Research Papers in Economics; and the National Bureau of Economic Research working paper series). We also examined references of included studies, forward and backward, to identify reviews our searches may have missed. Databases were searched for the time period ranging from January 1, 2005, to May 18, 2023. At least two independent reviewers screened titles and abstracts, then full texts, in duplicate to identify relevant citations. Any conflicts were reviewed by a third independent reviewer. The search strategy is available in Supplementary Material Appendix 1.

### Inclusion and exclusion criteria

All review types (e.g., umbrella, systematic, rapid, scoping, meta-analysis, meta-regression) were included. As a framework to select relevant policies, we used the World Health Organization Global Strategy to Reduce the Harmful Use of Alcohol’s target policy areas to reduce the consumption of alcohol (World Health Organization, 2010). We included population-level policies that seek to regulate the public availability of alcohol, such as establishing a minimum purchase age, regulating outlet density, and setting days/hours of sale; and policies related to the marketing of alcoholic beverages, such as regulating the volume of advertising from alcohol manufacturers, requiring plain packaging for alcohol and adding alcohol warning labels to alcohol packaging, establishing self-regulation of alcohol marketing, and advertising from government authorities to minimize harm of alcohol use. We excluded price and tax policies that seek to increase the price of alcohol products and incentivize the purchase of non-alcoholic beverages because we investigated this question in a companion umbrella review (Guindon et al., 2022). All measures of alcohol use (e.g., heavy drinking, problematic alcohol use, binge drinking, heavy alcohol use) were included as outcomes. We excluded reviews with a focus on low- or middle-income countries to ensure generalizability to Canada. Only reviews written in English or French published since January 2005 were included.

### Data extraction, risk of bias assessment, and data synthesis

At least two independent reviewers extracted review characteristics and results using a standardized data extraction form and assessed the risk of bias of each review. For each policy/intervention, the following study characteristics were extracted and summarized: review type (e.g., umbrella, meta-analysis), research question, competing interests and funding disclosure, study focus (e.g., effectiveness, cost-effectiveness, cost–benefit), outcome measures, population, search strategy, number of included studies with alcohol use as the outcome, quality/risk of bias assessment conducted, and main findings. We refrained from using the term “systematic” to describe review type to avoid having to categorize included reviews as “systematic” vs. “not systematic.” We followed the preferred reporting items for overviews of reviews (PRIOR) statement (Gates et al., 2022). In synthesizing results, we paid particular attention to the magnitudes of effects and possible modifiers such as sex, age, and socioeconomic status.

To assess the risk of bias of included reviews, we used ROBIS, a tool for assessing Risk Of Bias In Systematic reviews (Whiting et al., 2016). The tool covers four domains through which bias may be introduced into a systematic review: study eligibility criteria, identification and selection of studies, data collection and study appraisal, and synthesis and findings. An overall risk of bias is provided after interpretation of review limitations from the identified domains. We used the ROBIS domains to assess the risk of bias of umbrella reviews. We did not contact authors of included reviews if information was missing or unclear. When included reviews included both reviews and primary studies, we categorized them as umbrella/review and discussed them with umbrella reviews. A completed PRIOR Checklist is available in Supplementary Material Appendix 2.

## Results

The database search identified 4932 records after the removal of duplicate citations, from which 4858 were excluded based on the title/abstract screening and 46 were subsequently removed after a full-text screening, yielding 28 reviews (ten umbrella reviews (Anderson et al., 2009; Booth et al., 2008; Burton et al., 2017; Jackson et al., 2010; Knai et al., 2015; Martineau et al., 2013; Petticrew et al., 2017; Siegfried & Parry, 2019; Stockings et al., 2016; Stockwell, 2006) and 18 reviews of individual studies (Bryden et al., 2012; Campbell et al., 2009; Clarke et al., 2020; Esser & Jernigan, 2018; Gmel et al., 2016; Grube & Waiters, 2005; Hahn et al., 2010, 2012; Hassan & Shiu, 2018; Hastings et al., 2005; Joyce et al., 2023; Kilian et al., 2023; Middleton et al., 2010; Popova et al., 2009; Scholes-Balog et al., 2012; Sherk et al., 2018; Siegfried et al., 2014; Young et al., 2018)) that met all inclusion criteria; two additional reviews were identified from references of reviews identified by the database searches (Wilkinson & Room, 2009; Wilkinson et al., 2009) and one was published after the database search was completed (Manthey et al., 2024). For comprehensiveness, we also included a review of primary studies published in 2002 which fell outside our date inclusion criteria because we were unable to identify any that met our inclusion criteria (Wagenaar & Toomey, 2002).

**Fig. 1:**
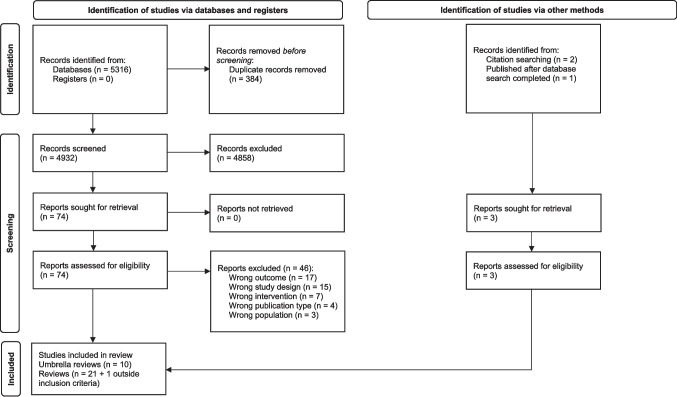
PRIOR flow diagram

A list of excluded studies and reasons for exclusion is available in Supplementary Material Appendix 3. Tables 1 and 2 present characteristics, main findings, and the risk of bias of included studies for policies aimed at addressing alcohol availability and alcohol marketing, respectively. Studies are presented by policy areas, in reverse chronological order, starting with umbrella reviews, followed by meta-analyses and narrative reviews. Figure 1 presents the PRIOR flow diagram. In Supplementary Material Appendix 4, we provide a list of all included studies for each policy domain that had a measure of alcohol use as an outcome, with the caveat that not all reviews clearly reported all included studies and their respective outcomes. We also provide, in Supplementary Tables A1–A8, a mapping of the primary studies contained within included reviews for each policy domain.

**Table 1:** Addressing alcohol availability: Study characteristics, main findings, and risk of bias

Review type; research question; competing interests; funding	Effectiveness/cost-effectiveness; outcome; population; search; no. of included studies	Quality/risk of bias assessment	Main findings	Risks of bias
**Minimum purchasing age**				
Stockings et al., 2016; *Lancet Psychiatry* - Umbrella review - Efficacy of interventions in young people with alcohol use - Competing interests: Disclosed (none reported) Funding: - Disclosed (none reported)	- Effectiveness; cost-effectiveness - Outcome: Alcohol use initiation or quantity, including problematic use - Population: Young people, aged 10–24 years old - Search: Jan 1990 to April 2015 - No. of studies included: 1 review (48 independent primary studies)	None. Level of evidence discussed according to number of systematic reviews, RCTs, etc	Results were suggestive, but inconclusive (about 40% of studies reported a significant effect) that raising the minimal legal drinking age reduced alcohol consumption *Magnitude of effect:* Mixed findings, small effect in reduction of problematic use	*Study eligibility criteria*: Unclear (eligibility criteria not described) *Identification and selection of studies:* High (too little information provided) *Data collection and study appraisal:* High (too little information provided) *Synthesis and findings*: Low Risk of bias: High
Martineau et al., 2013; *Prev Med* - Umbrella review - Population-level interventions to reduce alcohol-related harm - Competing interests: Disclosed (none reported) Funding: - National Institute for Health Research School for Public Health Research	- Effectiveness; cost-effectiveness - Outcome: Alcohol sales or consumption (specific outcome measures not clearly specified) - Population: General population - Search: Jan 2002 to Oct 2012 - No. of studies included: 2 reviews (48 independent primary studies)	AMSTAR I; assessment not provided; only broad quality scores (high, mid, low) provided	Consistent evidence that a higher minimum legal drinking age was associated with a reduction of alcohol consumption *Magnitude of effect*: Unclear	*Study eligibility criteria*: Low *Identification and selection of studies:* High (no duplicate study selection) *Data collection and study appraisal:* High (study data not all extracted in duplicate; limited data extraction table provided) *Synthesis and findings*: High (no published protocol; quality assessment not addressed in synthesis or discussion of results) Risk of bias: High
Jackson et al., 2010; *Report* (University of Sheffield) - Umbrella review/narrative review - Effectiveness of interventions to manage alcohol availability to reduce levels of consumption, alcohol misuse, related harm or social problems - Competing interests: Not disclosed Funding: - Centre for Public Health Excellence, National Institute for Health and Clinical Excellence	- Effectiveness; cost-effectiveness - Outcome: Alcohol consumption (consumption in the past 30 days) - Population: Adults and young people aged 10 years and above - Search: 2008 (from inception) - No. of studies included: 2 reviews; 1 primary study (49 independent primary studies)	A quality checklist for reviews was developed. A subjective cutoff score of 9 criteria fulfilled (of a total of 14) was deemed of higher quality. Assessment not provided; only 3 broad quality scores provided	In one systematic review, most studies (87%) reported that a higher minimum legal drinking age was associated with lower alcohol consumption, but 46% were not statistically significant. One primary study examining prevalence of alcohol use in high school seniors did not find a statistically significant change in prevalence *Magnitude of effect*: Unclear	*Study eligibility criteria*: Low *Identification and selection of studies:* Low *Data collection and study appraisal:* Low *Synthesis and findings*: Low Risk of bias: Low
Anderson et al., 2009; *Lancet* - Umbrella review - Effectiveness and cost-effectiveness of policies and programs to reduce the harm caused by alcohol - Competing interests: Disclosed (none reported) Funding: - Not disclosed	- Effectiveness; cost-effectiveness - Outcome: Not clearly reported - Population: Unclear - Search: Unclear - No. of studies included: 1 review (41 independent primary studies)	None. The quality of included studies was not assessed. The strength of the evidence was based on the type of study design of included studies: 1 = more than one systematic review; 2 = one systematic review; 3 = 2 or more randomized controlled trials; 4 = one randomized controlled trial; 5 = observational evidence; 6 = not assessed	Minimum drinking age laws were associated with a reduction in youth drinking *Magnitude of effect*: Unclear	*Study eligibility criteria*: Unclear (eligibility criteria not described) *Identification and selection of studies:* Unclear (too little information provided) *Data collection and study appraisal:* High (reviews not appraised for risk of bias) *Synthesis and findings*: High (potential biases not accounted for; findings incompletely reported) Risk of bias: High
Wagenaar & Toomey, 2002; *J Stud Alcohol* - Narrative review - To determine the overall effect of the age-21 minimum drinking age laws on youth alcohol use - Competing interests: Not disclosed Funding: - Not disclosed	- Effectiveness - Outcome: Alcohol consumption - Population: Youth, including college-age students - Search: 1960–1999 - No. of studies included: 48 independent primary studies	None. “Methodological quality” was assessed by examining three design components: (1) sampling design (nonprobability sampling vs higher quality probability sampling or census data); (2) study design (cross-sectional vs pre-post longitudinal); (3) some form of comparison group was used	From 48 studies, there was a statistically significant negative relationship between minimum legal drinking age and alcohol consumption in 27 (35%) of 78 analyses; significant findings were consistent between cross-sectional (8/21) and longitudinal evidence (19/57) *Magnitude of effect*: Unclear	*Study eligibility criteria*: High (unclear whether a protocol with pre-defined objectives and eligibility criteria was developed and followed) *Identification and selection of studies:* Low *Data collection and study appraisal:* High (steps to minimize error in data collection not described; risk of bias of primary studies not assessed using a formal tool) *Synthesis and findings*: High (synthesis only described quality of included studies and focused on statistical significance of findings) Risk of bias: High
**Days/hours of sale**				
Siegfried & Parry, 2019; *PLoS One* - Umbrella review - Effectiveness of policies to limit the availability of alcohol - Competing interests: Disclosed (GAPA, UNODC, WHO) Funding: - Drug Research Unit of the South African Medical Research Council	- Effectiveness; cost-effectiveness - Outcome: Alcohol consumption (not described further) - Population: General population - Search: Jan 2006–Jul 2017 - No. of studies included: 5 reviews (15 independent primary studies)	ROBIS; only broad scores (high, low, uncertain) presented	Only broad conclusions provided. There was “uncertain” evidence that licensing restrictions (including banning sales, and making changes to the hours, days and volumes of alcohol sales) were associated with lower alcohol use *Magnitude of effect*: Unclear	S*tudy eligibility criteria*: Low *Identification and selection of studies:* Low *Data collection and study appraisal:* High (extraction process not described; characteristics of included reviews not appropriately presented) *Synthesis and findings*: High (synthesis of results restricted to reviews at low risk of bias) Risk of bias: High
Burton et al., 2017; *Lancet* - Umbrella review/narrative rapid review - Effectiveness and cost-effectiveness of policies to reduce alcohol-related harms in England - Competing interests: Disclosed (none reported) Funding: - Review was commissioned by the Department of Health; resources were provided by Public Health England	- Effectiveness; cost-effectiveness - Outcome: Alcohol consumption - Population: Adults with some underage consumption data - Search: 2000 to 2016 - No. of studies included: 5 reviews/reports; 1 primary study (12 independent primary studies)	GRADE	Limited and mixed evidence about an association between hours of sale and alcohol consumption. Most of the evidence reported pertained to harm, not consumption *Magnitude of effect*: Unclear	*Study eligibility criteria*: Unclear (no pre-defined protocol; some inappropriate eligibility criteria) *Identification and selection of studies:* Low *Data collection and study appraisal:* High (study characteristics not provided; GRADE not designed to assess risk of bias) *Synthesis and findings*: Unclear (findings incompletely reported) Risk of bias: High
Stockings et al., 2016; *Lancet Psychiatry* - Umbrella review - Efficacy of interventions in young people with alcohol use - Competing interests: Disclosed (none reported) Funding: - Disclosed (none reported)	- Effectiveness; cost-effectiveness - Outcome: Alcohol use initiation or quantity, including problematic use - Population: Young people, aged 10–24 years old - Search: Jan 1990 to Apr 2015 - No. of studies included: 3 (1 umbrella review; 1 umbrella/narrative review; 1 book) (15 independent primary studies)	None. Level of evidence discussed according to number of systematic reviews, RCTs, etc	There was conflicting evidence on the effect of limiting hours of operation on alcohol consumption in young people. The effectiveness of restrictions depended on alcohol availability and hours of operation in surrounding areas *Magnitude of effect*: Unclear	*Study eligibility criteria*: Unclear (eligibility criteria not described) *Identification and selection of studies:* High (too little information provided) *Data collection and study appraisal:* High (too little Information provided) *Synthesis and findings*: Low Risk of bias: High
Martineau et al., 2013; *Prev Med* - Umbrella review - Population-level interventions to reduce alcohol-related harm - Competing interests: Disclosed (none reported) Funding: - National Institute for Health Research School for Public Health Research	- Effectiveness; cost-effectiveness - Outcome: Alcohol sales or consumption (specific outcome measures not clearly reported) - Population: General population - Search: Jan 2002 to Oct 2012 - No. of studies included: 4 reviews (18 independent primary studies)	AMSTAR I; only broad scores (high, mid, low) provided	Restricting days or hours of sale was consistently associated with lower alcohol consumption; increasing the days/hours of alcohol sale availability was associated with increased total alcohol consumption *Magnitude of effect*: Unclear	*Study eligibility criteria*: Low *Identification and selection of studies:* High (no duplicate study selection) *Data collection and study appraisal:* High (study data not all extracted in duplicate; limited data extraction table provided) *Synthesis and findings*: High (no published protocol; quality assessment not addressed in synthesis or discussion of results) Risk of bias: High
Jackson et al., 2010; *Report* (University of Sheffield) - Umbrella review/narrative review - Effectiveness of interventions to manage alcohol availability to reduce levels of consumption, alcohol misuse, related harm or social problems - Competing interests: Not disclosed Funding: - Centre for Public Health Excellence, National Institute for Health and Clinical Excellence	- Effectiveness; cost-effectiveness - Outcome: Alcohol consumption (consumption in the past 30 days) - Population: Adults and young people aged 10 years and above - Search: 2008 (from inception) - No. of studies included: 2 reviews; 6 primary studies (6 independent primary studies)	A quality checklist for reviews was developed. A subjective cutoff score of 9 criteria fulfilled (of a total of 14) was deemed of higher quality; only 3 broad quality scores provided	The UK Licensing Act 2003, that abolished set licensing hours in England and Wales, was not associated with increased alcohol consumption Other UK-specific studies and international evidence presented mixed findings One review pointed to one study which indicated that reducing licensed hours of sale was relatively low cost *Magnitude of effect*: Unclear	*Study eligibility criteria*: Low *Identification and selection of studies:* Low *Data collection and study appraisal:* Low *Synthesis and findings*: Low Risk of bias: Low
Anderson et al., 2009; *Lancet* - Umbrella review - Effectiveness and cost-effectiveness of policies and programs to reduce the harm caused by alcohol - Competing interests: Disclosed (none reported) Funding: - Not disclosed	- Effectiveness; cost-effectiveness - Outcome: Not clearly reported - Population: Unclear - Search: Unclear - No. of studies included: 2 (1 book; 1 report) (unclear no. of independent primary studies)	None. The quality of included studies was not assessed. The strength of the evidence was based on the type of study design of included studies: 1 = more than one systematic review; 2 = one systematic review; 3 = 2 or more randomized controlled trials; 4 = one randomized controlled trial; 5 = observational evidence; 6 = not assessed	There was some review-level evidence supporting the association between reduction of days/hours of alcohol availability and reduction of alcohol consumption *Magnitude of effect*: Unclear	S*tudy eligibility criteria*: Unclear (eligibility criteria not described) *Identification and selection of studies:* Unclear (too little information provided) *Data collection and study appraisal:* High (reviews not appraised for risk of bias) *Synthesis and findings*: High (potential biases not accounted for; findings incompletely reported) Risk of bias: High
Kilian et al., 2023; *EClinicalMedicine* - Narrative review/meta-analysis - Effectiveness of alcohol control measures in reducing alcohol consumption - Competing interests: Disclosed (NABCA) Funding: - National Institute of Alcohol Abuse and Alcoholism of the US National Institutes of Health	- Effectiveness - Outcome: Alcohol consumption (day-specific alcohol consumption, per capita sales, past month alcohol use and binge drinking) - Population: General population - Search: Jan 2000 to Sept 2022 - No. of studies included: 10 independent primary studies	Newcastle–Ottawa Scale; only broad scores presented	Restrictions on temporal availability (days/hours of sale) were associated with a statistically significant decrease in consumption, driven by decreases in beer and wine consumption *Magnitude of effect*: Restriction of alcohol sales by 1 day was associated with 3.6% (95%CI − 5.1, − 2.2) lower alcohol consumption	*Study eligibility criteria*: Low *Identification and selection of studies:* Low *Data collection and study appraisal:* Low *Synthesis and findings*: Low Risk of bias: Low
Sherk et al., 2018; *J Stud Alcohol Drugs* - Narrative review/meta-analysis - Physical availability of take-away alcohol, determined by hours of sale and outlet density, impact on alcohol consumption - Competing interests: Systembolaget (the Swedish government alcohol monopoly which reports to the Ministry of Health) Funding: - Systembolaget	- Effectiveness - Outcome: Alcohol consumption (per capita, total, and beverage-specific) - Population: unclear - Search: December 2015 (from inception) - No. of studies included: 7 independent primary studies	None	An additional day of sale was associated with small increases in per capita alcohol consumption. Increasing hours of sales was also associated with increases in consumption; however, evidence is limited *Magnitude of effect*: One additional day of sale (Sunday/Saturday) was associated with per capita consumption increases of 3.4% (95% CI: 2.7, 4.1) for total alcohol, 5.3% (3.2, 7.4) for beer, 2.6% (1.8, 3.5) for wine, and 2.6% (2.1, 3.2) for spirits	*Study eligibility criteria*: Low *Identification and selection of studies*: Low *Data collection and study appraisal*: High (no risk of bias assessment) *Synthesis and findings*: Low Risk of bias: High
Bryden et al., 2012; *Health & Place* - Narrative review - Community-level alcohol availability and marketing influence on alcohol use - Competing interests: Not disclosed Funding: - Disclosed (none reported)	- Effectiveness - Outcome: Alcohol use (community-level use, prevalence, quantity or frequency, harmful use) - Population: General population - Search: October 2010 (from inception) - No. of studies included: 3 independent primary studies	Effective Public Health Practice Project (EPHPP) Quality Assessment Tool for Quantitative Studies; only broad scores (weak, moderate, strong) provided	Restricting alcohol sale days and hours may be associated with a reduction in alcohol use *Magnitude of effect*: Unclear	*Study eligibility criteria*: Low *Identification and selection of studies:* Low *Data collection and study appraisal:* Unclear (limited information on risk of bias assessment) *Synthesis and findings*: Low Risk of bias: Low
Hahn et al., 2010; *Am J Prev Med* - Narrative review - Effect of changing hours of sale at on- or off-premise outlets on excessive alcohol consumption - Competing interests: Disclosed (none reported) Funding: - Disclosed (none reported)	- Effectiveness - Outcome: Binge drinking, heavy drinking, per capita alcohol consumption - Population: Unclear - Search: Feb 2008 (from inception) - No. of studies included: 4 independent primary studies	Community guide systematic review protocol	There were no consistent effects on alcohol consumption (including excessive) of increasing hours of sales *Magnitude of effect:* Unclear	*Study eligibility criteria*: Low *Identification and selection of studies:* Low *Data collection and study appraisal:* Low *Synthesis and findings*: Low Risk of bias: Low
Middleton et al., 2010; *Am J Prev Med* - Narrative review - Effects of reducing or increasing days/hours of sale on alcohol consumption - Competing interests: Not disclosed Funding: - Disclosed (none reported)	- Effectiveness; cost-effectiveness - Outcome: Binge drinking, heavy drinking, per capita alcohol consumption - Population: Unclear - Search: February 2008 (from inception) - No. of studies included: 8 independent primary studies (1 economic evaluation)	Quality assessment completed using Community Guide systematic review protocol; six categories of threats to validity: study population and intervention descriptions, sampling, exposure and outcome measurement, data analysis, interpretation of results (including follow-up, bias, and confounding), and other. Each study is categorized as having good, fair, or limited quality of execution based on the number of limitations noted. Assessment of primary studies not reported	Increasing days of sale by allowing previously banned alcohol sales on either Saturdays or Sundays was associated with increased excessive alcohol consumption; decreasing days of sale for spirits and wine but not beer was associated with a substitution of beer for wine and spirits One modelling study concluded that restricting alcohol sales for a 24-h period over the weekend was very cost effective *Magnitude of effect:* Effect sizes may have been large enough to be meaningful	*Study eligibility criteria*: Low *Identification and selection of studies:* Low *Data collection and study appraisal:* Low *Synthesis and findings*: Low Risk of bias: Low
Popova et al., 2009, *Alcohol Alcohol* - Narrative review - How the availability of alcohol (hours, days, and outlet density) impacts alcohol consumption and damage - Competing interests: Not disclosed Funding: - Department of Health Promotion and Protection, Nova Scotia; Ontario Ministry of Health and Long-Term Care	- Effectiveness - Outcome: Alcohol consumption (per capita sales or total volume) and drinking patterns - Search: Jan 2000 to Dec 2008 - No. of studies included: 3 independent primary studies	None	Later hours of sale were associated with increased sales of high alcohol content beer, wine, and distilled spirits. In Sweden, opening alcohol outlets on Saturdays in a small region and later the whole of Sweden was associated with increases in sales *Magnitude of effect*: Effect sizes may have been large enough to be meaningful	*Study eligibility criteria*: Low *Identification and selection of studie*s: Low *Data collection and study appraisal:* High (no risk of bias assessment; study characteristics of primary studies not provided) *Synthesis and findings*: High (not all included studies were reported in the synthesis and biases in primary studies were not sufficiently addressed) Risk of bias: High
**Alcohol outlet density**				
Burton et al., 2017; *Lancet* - Umbrella review/narrative rapid review - Effectiveness and cost-effectiveness of policies to reduce alcohol-related harms in England - Competing interests: Disclosed (none reported) Funding: - Review was commissioned by the Department of Health; resources were provided by Public Health England	- Effectiveness; cost-effectiveness - Outcome: Alcohol consumption - Population: Adults with some underage consumption data - Search: 2000 to 2016 - No. of studies included: 7 reviews (28 independent primary studies)	GRADE	Limited and mixed evidence about an association between outlet density and alcohol consumption. Most of the evidence reported pertained to harm, not consumption One review indicated that reducing on-trade outlet opening hours targeting the most densely populated areas with simultaneous enforcement was cost-effective *Magnitude of effect:* Unclear	*Study eligibility criteria*: Unclear (no pre-defined protocol; some inappropriate eligibility criteria) *Identification and selection of studies:* Low *Data collection and study appraisal:* High (study characteristics not provided; GRADE not designed to assess risk of bias) *Synthesis and findings*: Unclear (findings incompletely reported) Risk of bias: High
Stockings et al., 2016; *Lancet Psychiatry* - Umbrella review - Efficacy of interventions in young people with alcohol use - Competing interests: Disclosed (none reported) Funding: - Disclosed (none reported)	- Effectiveness; cost-effectiveness - Outcome: Alcohol use initiation or quantity, including problematic use - Population: young people, aged 10–24 years - Search: Jan 1990 to Apr 2015 - No. of studies included: 1 book (unclear no. of independent primary studies)	None. Level of evidence discussed according to number of systematic reviews, RCTs, etc	Restrictions to the number of outlets where alcohol is allowed to be sold can reduce young people’s access to alcohol *Magnitude of effect:* Unclear	S*tudy eligibility criteria*: Unclear (eligibility criteria not described) *Identification and selection of studies:* High (too little information provided) *Data collection and study appraisal:* High (too little information provided) *Synthesis and findings*: Low Risk of bias: High
Martineau et al., 2013; *Prev Med* - Umbrella review - Population-level interventions to reduce alcohol-related harm - Competing interests: Disclosed (none reported) Funding: - National Institute for Health Research School for Public Health Research	- Effectiveness; cost-effectiveness - Outcome: Alcohol sales or consumption (specific outcome measures not clearly specified) - Population: General population - Search: Jan 2002 to Oct 2012 - No. of studies included: 1 umbrella review (11 independent primary studies)	AMSTAR I; only broad scores (high, mid, low) provided	Increasing outlet density was associated with increasing alcohol consumption *Magnitude of effect*: Unclear	S*tudy eligibility criteria*: Low *Identification and selection of studies:* High (no duplicate study selection) *Data collection and study appraisal:* Low *Synthesis and findings*: High (no published protocol; quality assessment not addressed in synthesis or discussion of results) Risk of bias: High
Jackson et al., 2010; *Report* (University of Sheffield) - Umbrella review/narrative review - Effectiveness of interventions to manage alcohol availability to reduce levels of consumption, alcohol misuse, related harm, or social problems - Competing interests: Not disclosed Funding: - Centre for Public Health Excellence, National Institute for Health and Clinical Excellence	- Effectiveness; cost-effectiveness - Outcome: Alcohol consumption (consumption in the past 30 days) - Population: Adults and young people aged 10 years and above - Search: 2008 (from inception) - No. of studies included: 2 reviews; 11 primary studies (11 independent primary studies)	A quality checklist for reviews was developed. A subjective cutoff score of 9 criteria fulfilled (of a total of 14) was deemed of higher quality; only 3 broad quality scores provided	There was a positive association between outlet density and alcohol consumption in adults and young people across different study designs and measures of consumption. One US study found that neighbourhood density, not individual distance to outlets, may be associated with consumption *Magnitude of effect*: Unclear	*Study eligibility criteria*: Low *Identification and selection of studies:* Low *Data collection and study appraisal:* Low *Synthesis and findings*: Low Risk of bias: Low
Anderson et al., 2009; *Lancet* - Umbrella review - Effectiveness and cost-effectiveness of policies and programs to reduce the harm caused by alcohol - Competing interests: Disclosed (none reported) Funding: - Not disclosed	- Effectiveness; cost-effectiveness - Outcome: Unclear - Population: Unclear - Search: Unclear - No. of studies included: 1 review; 1 primary study (1 independent primary study)	None. The quality of included studies was not assessed. The strength of the evidence was based on the type of study design of included studies: 1 = more than one systematic review; 2 = one systematic review; 3 = 2 or more randomized controlled trials; 4 = one randomized controlled trial; 5 = observational evidence; 6 = not assessed	An increased density of alcohol outlets was associated with increased amounts of alcohol consumption among young people The effects of reducing access to retail outlets for specified periods of the week have the potential to be very cost-effective *Magnitude of effect*: Unclear	*Study eligibility criteria*: Unclear (eligibility criteria not described) *Identification and selection of studies:* Unclear (too little information provided) *Data collection and study appraisal:* High (reviews not appraised for risk of bias) *Synthesis and findings*: High (potential biases not accounted for; findings incompletely reported) Risk of bias: High
Sherk et al., 2018; *J Stud Alcohol Drugs* - Narrative review/meta-analysis - Physical availability of take-away alcohol, determined by hours of sale and outlet density, impact on alcohol consumption - Competing interests: Systembolaget Funding: - Systembolaget	- Effectiveness - Outcome: Alcohol consumption (per-capita, total and beverage-specific) - Population: Unclear - Search: Dec 2015 (from inception) - No. of studies included: 4 independent primary studies	None. Study quality assessed based on the strength of study design: Tier 1: Pre-, post-natural experimental with simultaneous control observations Tier 2: Pre-, post-natural experiments with no control observations Tier 3: All other studies (e.g., cross-sectional)	Two of three studies in Canada reported significant positive associations between alcohol outlet density and alcohol consumption. The third reported a non-significant increase. One study in the US reported a significant positive association between alcohol outlet density and weekly consumption *Magnitude of effect*: Canadian studies reported outlet elasticities of alcohol between 0.07 and 0.19. In the US, a one standard deviation increase in outlet density was associated with a 7% and an 11% increase in consumption for men and for women, respectively	*Study eligibility criteria*: Low *Identification and selection of studies:* Low *Data collection and study appraisal:* Unclear (no risk of bias assessment) *Synthesis and findings*: Low Risk of bias: Low
Gmel et al., 2016; *Drug Alcohol Rev* - Narrative review - Outlet density and associated alcohol-related outcomes - Competing interests: Not disclosed Funding: - Not disclosed	- Effectiveness - Outcome: Alcohol-related outcomes, including consumption (not described) - Population: Non-specified general adult population - Search: Jan 2009–Oct 2014 - No. of studies included: 10 independent primary studies	None	There was little evidence that outlet density was strongly associated with alcohol use. Effects were inconsistent with regard to binge drinking or consumption volume *Magnitude of effect*: Unclear	*Study eligibility criteria*: Low *Identification and selection of studies:* High (grey literature not searched) *Data collection and study appraisal:* High (too little information provided) *Synthesis and findings*: High (all included studies not mentioned in the synthesis) Risk of bias: High
Bryden et al., 2012; *Health Place* - Narrative review - Community-level alcohol availability and marketing influence on alcohol use - Competing interests: Not disclosed Funding: - Disclosed (none reported)	- Effectiveness - Outcome: Alcohol use (community-level use, prevalence, quantity or frequency, harmful use) - Population: General population - Search: Oct 2010 (from inception) - No. of studies included: 13 independent primary studies	Effective Public Health Practice Project (EPHPP) Quality Assessment Tool for Quantitative Studies; only broad scores (weak, moderate, strong) provided	There was mixed evidence on the effectiveness of reducing alcohol outlet density to reduce consumption, though this might be more effective among adolescents *Magnitude of effect*: Unclear	*Study eligibility criteria*: Low *Identification and selection of studies:* Low *Data collection and study appraisal:* Unclear (no information on risk of bias assessment) *Synthesis and findings*: Low Risk of bias: Low
Campbell et al., 2009; *Am J Prev Med* - Narrative review - Effect of alcohol outlet density on excessive alcohol consumption - Competing interests: Disclosed (none reported) Funding: - Disclosed (none reported)	- Effectiveness; cost-effectiveness - Outcome: Alcohol consumption (per capita consumption of alcohol, binge drinking) - Population: General population, excluding college students - Search: Nov 2006 (from inception) - No. of studies included: 9 independent primary studies	Quality assessment completed using Community Guide systematic review protocol; six categories of threats to validity: study population and intervention descriptions, sampling, exposure and outcome measurement, data analysis, interpretation of results (including follow-up, bias, and confounding), and other. Each study is categorized as having good, fair, or limited quality of execution based on the number of limitations noted. Assessment of primary studies not reported	Consistent evidence in cross-sectional and time series analysis of a positive association between outlet density and alcohol consumption. The association appeared to be greater for off-premises outlets than on-premises *Magnitude of effect*: On- and off-premises aggregated, mean elasticity 0.27; on-premises only, mean-elasticity 0.25; off-premises only 2.46	*Study eligibility criteria*: Low *Identification and selection of studies:* Low *Data collection and study appraisal:* Low *Synthesis and findings*: Unclear (not enough information provided regarding protocol or synthesis) Risk of bias: Low
Popova et al., 2009 *Alcohol Alcohol* - Narrative review - Impact of alcohol availability on consumption and damage - Competing interests: Not disclosed Funding: - Department of Health Promotion and Protection, Nova Scotia; Ontario Ministry of Health and Long-Term Care	- Effectiveness - Outcome: Overall alcohol consumptions and drinking patterns - Population: General population - Search: Jan 2000–Dec 2008 - No. of studies included: 11 independent primary studies	None	Evidence from most studies support an association between higher alcohol outlet density and increased alcohol consumption *Magnitude of effect*: Unclear	*Study eligibility criteria*: Low *Identification and selection of studies*: Low *Data collection and study appraisal:* High (no risk of bias assessment; study characteristics of primary studies not provided) *Synthesis and findings*: High (not all included studies were reported in the synthesis; biases in primary studies were not sufficiently addressed) Risk of bias: High
**Retail privatization, monopolization**				
Martineau et al., 2013; *Prev Med* - Umbrella review - Population-level interventions to reduce alcohol-related harm - Competing interests: Disclosed (none reported) Funding: - National Institute for Health Research School for Public Health Research	- Effectiveness; cost-effectiveness - Outcome: Alcohol sales or consumption (specific outcome measures not clearly specified) - Population: General population - Search: Jan 2002 to Oct 2012 - No. of studies included: 2 reviews (15 independent primary studies)	AMSTAR I; only broad scores (high, mid, low) provided	Two reviews rated “low quality” concluded that privatization of government monopolies had consistently led to increased excessive alcohol consumption. One review rated “low quality” concluded re-monopolization led to lower excessive alcohol consumption *Magnitude of effect*: Unclear	*Study eligibility criteria*: Low *Identification and selection of studies:* High (no duplicate study selection) *Data collection and study appraisal:* High (study data not all extracted in duplicate; limited data extraction table provided) *Synthesis and findings*: High (no published protocol; quality assessment not addressed in synthesis or discussion of results) Risk of bias: High
Anderson et al., 2009; *Lancet* - Umbrella review - Effectiveness and cost-effectiveness of policies and programs to reduce the harm caused by alcohol - Competing interests: Disclosed (none reported) Funding: - Not disclosed	- Effectiveness; cost-effectiveness - Outcome: Unclear - Population: Unclear - Search: Unclear - No. of studies included: 0 review, 1 primary study	None. The quality of included studies was not assessed. The strength of the evidence was based on the type of study design of included studies: 1 = more than one systematic review; 2 = one systematic review; 3 = 2 or more randomized controlled trials; 4 = one randomized controlled trial; 5 = observational evidence; 6 = not assessed	Government monopolies were found to be “effective” at reducing alcohol use. Relative to alcohol retail systems of private sellers, government monopolies tended to have fewer stores, which were open for shorter hours *Magnitude of effect*: Unclear	*Study eligibility criteria*: Unclear (eligibility criteria not described) *Identification and selection of studies:* Unclear (too little information provided) *Data collection and study appraisal:* High (reviews not appraised for risk of bias) *Synthesis and findings*: High (potential biases not accounted for; findings incompletely reported) Risk of bias: High
Hahn et al., 2012; *Am J Prev Med* - Narrative review - Effects of alcohol retail privatization on excessive alcohol consumption and related harms - Competing interests: Not disclosed Funding: - Disclosed (none reported)	- Effectiveness; cost-effectiveness - Outcome: Excessive alcohol consumption, proxied by per capita alcohol consumption; self-reported alcohol consumption - Population: General population - Search: Dec 2010 (from inception) - No. of studies included: 12 distinct privatization events, assessed in 17 studies, reported in 15 publications; 1 economic evaluation	Quality assessment completed using Community Guide systematic review protocol; six categories of threats to validity: study population and intervention descriptions, sampling, exposure and outcome measurement, data analysis, interpretation of results (including follow-up, bias, and confounding), and other. Each study is categorized as having good, fair, or limited quality of execution based on the number of limitations noted. Assessment of primary studies not reported	Privatization of retail alcohol sales was consistently associated with a substantial increase in per capita sales of the privatized beverages; re-monopolization in Sweden was associated with a decrease in alcohol-related harms (alcohol use as an outcome was not discussed) One modelling study estimated healthcare and law enforcement costs and costs of lost productivity in the event all Canadian provinces and territories were to privatize alcohol sales, and concluded that costs were substantially greater than the tax and mark-up revenue gained from increased sales associated with privatization; benefits, however, were not documented *Magnitude of effect*: 44% median (IQR 5, 123) increase in per capita sales of privatized beverages in locations that privatized retail alcohol sales; during the same time period, sales of non-privatized alcoholic beverages decreased by a median of 2% (IQR 0, 7)	*Study eligibility criteria*: Low *Identification and selection of studies:* Low (search terms not available; excluded unpublished reports) *Data collection and study appraisal:* Low (risk of bias procedure not outlined; primary characteristics of included studies not provided) *Synthesis and findings*: Low Risk of bias: Low

*AMSTAR* A MeaSurement Tool to Assess systematic Reviews, *GAPA* Global Alcohol Policy Alliance, *MLDA* minimum legal drinking age, *NABCA* National Alcohol Beverage Control Association, *RCT* randomized controlled trial, *ROBIS* Risk Of Bias In Systematic reviews, *UNODC* United Nations Office on Drugs and Crime, *WHO* World Health Organization

**Table 2:** Addressing alcohol marketing: Study characteristics, main findings, and risk of bias

Review type; research question; competing interests; funding	Study type; outcome; population; search; no. of included studies	Quality/risk of bias assessment	Main findings	Risks of bias
**Self-regulation**				
Anderson et al., 2009; *Lancet* - Umbrella review - Effectiveness and cost-effectiveness of policies and programs to reduce the harm caused by alcohol - Competing interests: Disclosed (none reported) Funding: - Not disclosed	- Effectiveness; cost-effectiveness - Outcome: Not clearly reported - Population: Unclear - Search: Unclear - No. of studies included: 1 primary study	None. The quality of included studies was not assessed. The strength of the evidence was based on the type of study design of included studies: 1 = more than one systematic review; 2 = one systematic review; 3 = 2 or more randomized controlled trials; 4 = one randomized controlled trial; 5 = observational evidence; 6 = not assessed	“No evidence for effectiveness. Studies show that self-regulation does not prevent types of marketing that can affect young people.” Conclusion not supported by the evidence presented *Magnitude of effect*: Unclear	*Study eligibility criteria*: Unclear (eligibility criteria not described) *Identification and selection of studies:* Unclear (too little information provided) *Data collection and study appraisal:* High (reviews not appraised for risk of bias) *Synthesis and findings*: High (potential biases not accounted for; findings incompletely reported) Risk of bias: High
Booth et al., 2008; *Report* (University of Sheffield) - Umbrella review - The effect of industry self-regulation of alcohol advertising on alcohol consumption - Competing interests: Disclosed (none reported) Funding: - Policy Research Program, Department of Health	- Effectiveness - Outcome: Population and individual alcohol consumption, intention to consume, and substitution - Population: Underage drinkers, youth adult binge drinkers, harmful/heavy drinkers, low income - Search: 2000–2008 - No. of studies included: 0	None. Bradford Hill’s principles were used as an aid for examining causation	There was no evidence found for or against self-regulation of alcohol advertising *Magnitude of effect*: Unclear	*Study eligibility criteria*: Low *Identification and selection of studies:* High (limited search; limited details on screening) *Data collection and study appraisal:* High (data extraction not described; no quality assessment) *Synthesis and findings*: Unclear (no published protocol; too little information provided) Risk of bias: High
**Advertising from government authorities to reduce alcohol use**				
Stockings et al., 2016; *Lancet Psychiatry* - Umbrella review - Efficacy of interventions in young people with alcohol use - Competing interests: Disclosed (none reported) Funding: - Disclosed (none reported)	- Effectiveness; cost-effectiveness - Outcome: Alcohol use (initiation or quantity of substance used), including problematic use (heavy use that may cause harm to self or others) - Population: Young people (10–24 years old) - Search: Jan 1990 to Apr 2015 - No. of studies included: 1 umbrella review (unclear no. of independent primary studies)	None. Level of evidence discussed according to number of systematic reviews, RCTs, etc	Findings were mixed and more evidence was required to assess effectiveness of mass media on youth consumption *Magnitude of effect*: Unclear	*Study eligibility criteria*: Unclear (eligibility criteria not described) *Identification and selection of studies:* High (too little information provided) *Data collection and study appraisal:* High (too little information provided) *Synthesis and findings*: Low Risk of bias: High
Martineau et al., 2013; *Prev Med* - Umbrella review - Population-level interventions to reduce alcohol-related harm - Competing interests: Disclosed (none reported) Funding: - National Institute for Health Research School for Public Health Research	- Effectiveness; cost-effectiveness - Outcome: Alcohol sales or consumption (specific outcome measures not clearly specified) - Population: General population - Search: Jan 2002 to Oct 2012 - No. of studies included: 3 reviews (unclear no. of independent primary studies)	AMSTAR I; only broad scores (high, mid, low) provided	Two meta-analyses rated “low quality” and one review rated “medium quality” concluded that mass media campaigns were associated with reduced alcohol use *Magnitude of effect*: Unclear	*Study eligibility criteria*: Low *Identification and selection of studies:* High (no duplicate study selection) *Data collection and study appraisal:* High (study data not all extracted in duplicate; limited data extraction table provided) *Synthesis and findings*: High (no published protocol; quality assessment not addressed in synthesis or discussion of results) Risk of bias: High
Anderson et al., 2009; *Lancet* - Umbrella review - Effectiveness and cost-effectiveness of policies and programs to reduce the harm caused by alcohol - Competing interests: Disclosed (none reported) Funding: - Not disclosed	- Effectiveness; cost-effectiveness - Outcome: Alcohol consumption (effectiveness and cost-effectiveness) - Population: Unclear - Search: Unclear - No. of studies included: 1 book (unclear no. of independent primary studies)	None. The quality of included studies was not assessed. The strength of the evidence was based on the type of study design of included studies: 1 = more than one systematic review; 2 = one systematic review; 3 = two or more randomized controlled trials; 4 = one randomized controlled trial; 5 = observational evidence; 6 = not assessed	Government advertising against alcohol (public information campaigns and counteradvertising) was not found to be effective at reducing alcohol *Magnitude of effect*: Unclear	*Study eligibility criteria*: Unclear (eligibility criteria not described) *Identification and selection of studies:* Unclear (too little information provided) *Data collection and study appraisal:* High (reviews not appraised for risk of bias) *Synthesis and findings*: High (potential biases not accounted for; findings incompletely reported) Risk of bias: High
Booth et al., 2008; *Report* (University of Sheffield) - Umbrella review - What is the effect of promotion (advertising) on alcohol consumption? - Competing interests: Disclosed (none reported) Funding: - Policy Research Program, Department of Health	- Effectiveness - Outcome: Population and individual alcohol consumption, intention to consume, and substitution of alcohol - Underage drinkers, youth adult binge drinkers, harmful/heavy drinkers, low income - Search: 2000–2008 - No. of studies included: 0 review, 7 primary studies	None. Bradford Hill’s principles were used as an aid for examining causation	Some evidence suggested that counteradvertising may have influenced youth decision-making, but generally counteradvertising was not considered to be as effective as commercial advertising *Magnitude of effect*: Unclear	*Study eligibility criteria*: Low *Identification and selection of studies:* High (limited search; limited details on screening) *Data collection and study appraisal:* High (data extraction not described; no quality assessment) *Synthesis and findings*: Unclear (no published protocol; too little information provided) Risk of bias: High
Young et al., 2018; *Alcohol Alcohol* - Narrative review - Effectiveness of mass media messages on alcohol consumption - Competing interests: Disclosed (none reported) Funding: - UK National Institute for Health Research Public Health Research	- Effectiveness - Outcome: Alcohol consumption (not clearly reported) - Population: Adults, young people and/or their parents, or pregnant women - Search: Jul 2016 (from inception) - No. of studies included: 13 primary studies	Effective Public Health Practice Project (EPHPP) Quality Assessment Tool for Quantitative Studies; only broad scores (weak, moderate, strong) provided	Although certain studies found a correlation between alcohol campaigns and reducing alcohol consumption, those are outnumbered by stronger studies that found no significant difference *Magnitude of effect*: Unclear	*Study eligibility criteria*: Low *Identification and selection of studies:* High (screening not done in duplicate) *Data collection and study appraisal:* Low *Synthesis and findings*: Low Risk of bias: Low
Bryden et al., 2012; *Health & Place* - Narrative review - Community-level alcohol availability and marketing influence on alcohol use - Competing interests: Not disclosed Funding: - Disclosed (none reported)	- Effectiveness - Outcome: Community-level alcohol use (prevalence, quantity, frequency, harmful use) - Population: Adults - Search: Inception to October 2010 - No. of studies included: 2 primary studies	Effective Public Health Practice Project (EPHPP) Quality Assessment Tool for Quantitative Studies; only broad scores (weak, moderate, strong) provided	Two studies of weak quality found a nonsignificant association between mass media campaigns and alcohol consumption *Magnitude of effect*: Unclear	*Study eligibility criteria*: Low *Identification and selection of studies:* Low *Data collection and study appraisal:* Unclear (insufficient information on number of reviewers) *Synthesis and findings*: Low Risk of bias: Low
**Regulating the volume of advertising from alcohol manufacturers**				
Siegfried & Parry, 2019; *PLoS One* - Umbrella review - Effectiveness of policies to limit the availability of alcohol - Competing interests: Disclosed (GAPA, UNODC, WHO) Funding: - Drug Research Unit of the South African Medical Research Council	- Effectiveness; cost-effectiveness - Outcome: Alcohol consumption (not described further) - Population: General population - Search: Jan 2006–Jul 2017 - No. of studies included: 1 review (3 independent primary studies)	ROBIS; only broad scores (high, low, or uncertain) presented	Only broad conclusions provided. The evidence was uncertain, but advertising restrictions were possibly beneficial *Magnitude of effect*: Unclear	*Study eligibility criteria*: Low *Identification and selection of studies:* Low *Data collection and study appraisal:* High (extraction process not described; included reviews’ characteristics not appropriately presented) *Synthesis and findings*: High (synthesis of results restricted to reviews at low risk of bias) Risk of bias: High
Burton et al., 2017; *Lancet* - Umbrella review/narrative rapid review - Effectiveness and cost-effectiveness of policies to reduce alcohol-related harms in England - Competing interests: Disclosed (none reported) Funding: - Review was commissioned by the Department of Health; resources were provided by Public Health England	- Effectiveness; cost-effectiveness - Outcome: Alcohol consumption - Population: Adults with some underage consumption data - Search: 2000 to 2016 - No. of studies included: 2 reviews; 3 primary economic evaluations (6 independent primary studies)	GRADE	Modelling studies have demonstrated that complete advertising bans were more effective and cost-effective than partial bans. Empirical evidence was sparse due to rare implementation of comprehensive bans *Magnitude of effect*: Unclear	*Study eligibility criteria*: Unclear (no pre-defined protocol; some inappropriate eligibility criteria) *Identification and selection of studies:* Low *Data collection and study appraisal:* High (study characteristics not provided; GRADE not designed to assess risk of bias) *Synthesis and findings*: Unclear (findings incompletely reported) Risk of bias: High
Petticrew et al., 2017; *J Epidemiol Community Health* - Umbrella review - Effectiveness of alcohol advertising restrictions on reducing alcohol consumption - Competing interests: Disclosed (none reported) Funding: - Medical Research Council Methods Research Programme	- Effectiveness - Outcome: Alcohol consumption (not clearly reported) - Population: Unclear - Search: Unclear - No. of studies included: 1 review (3 independent primary studies)	None	One review concluded that there was a lack of robust evidence for or against recommending the implementation of alcohol advertising restrictions *Magnitude of effect*: Unclear	*Study eligibility criteria*: Unclear (eligibility criteria not described) *Identification and selection of studies:* High (insufficient information on search strategy) *Data collection and study appraisal:* Unclear (insufficient information on number of reviewers) *Synthesis and findings*: Unclear (no published protocol; too little information provided) Risk of bias: Unclear
Stockings et al., 2016; *Lancet Psychiatry* - Umbrella review - Efficacy of interventions in young people with alcohol use - Competing interests: Disclosed (none reported) Funding: - Disclosed (none reported)	- Effectiveness; cost-effectiveness - Outcome: Alcohol use (initiation or quantity of substance used), including problematic use (heavy use that may cause harm to self or others) - Population: Young people (10–24 years old) - Search: Jan 1990 to Apr 2015 - No. of studies included: 2 reviews (3 independent primary studies)	None. Level of evidence discussed according to number of systematic reviews, RCTs, etc	A review of 4 studies (one randomized controlled trial and three interrupted time series) reported that the evidence was inconsistent on whether banning alcohol advertising reduced alcohol use in young people *Magnitude of effect:* Mixed findings, small effect in reduction of problematic use	*Study eligibility criteria*: Unclear (eligibility criteria not described) *Identification and selection of studies:* High (too little information provided) *Data collection and study appraisal:* High (too little information provided) *Synthesis and findings*: Low Risk of bias: High
Knai et al., 2015; *Addiction* - Umbrella review - Effectiveness of restricting advertising and marketing on reducing alcohol consumption - Competing interests: Disclosed (none reported) Funding: - Department of Health Policy Research Programme	- Effectiveness - Outcome: Alcohol consumption (specific outcome measures not clearly specified) - Population: General population of any age group - Search: Dec 2013 (unclear whether from inception) - No. of studies included: 0	None. The quality of included studies was not assessed. The strength of the evidence was based on the type of study design of included studies: 1 = systematic reviews; 2 = reviews with three core criteria; i.e., evidence of comprehensive search, clear selection (inclusion/exclusion) criteria and process of quality assessment of papers reviewed; 3 = reviews not meeting the criteria of level 2	There were no reviews directly examining the association between removing advertising near schools and alcohol consumption *Magnitude of effect*: Unclear	*Study eligibility criteria*: Low *Identification and selection of studies:* High (too little information provided) *Data collection and study appraisal:* High (limited data extraction table provided) *Synthesis and findings*: Unclear (no published protocol; limited data on synthesis methods) Risk of bias: High
Martineau et al., 2013; *Prev Med* - Umbrella review - Population-level interventions to reduce alcohol-related harm - Competing interests: Disclosed (none reported) Funding: - National Institute for Health Research School for Public Health Research	- Effectiveness; cost-effectiveness - Outcome: Alcohol sales or consumption (specific outcome measures not clearly specified) - Population: General population - Search: Jan 2002 to Oct 2012 - No. of studies included: 1 umbrella review (4 independent primary studies)	AMSTAR I; only broad scores (high, mid, low) provided	One umbrella review found there was insufficient and inconclusive evidence on the effectiveness of restricting advertising *Magnitude of effect*: Unclear	*Study eligibility criteria*: Low *Identification and selection of studies:* High (no duplicate study selection) *Data collection and study appraisal:* High (study data not all extracted in duplicate; limited data extraction table provided) *Synthesis and findings*: High (no published protocol; quality assessment not addressed in synthesis or discussion of results) Risk of bias: High
Jackson et al., 2010; *Report* (University of Sheffield) - Umbrella review/narrative review - Effectiveness of interventions to manage alcohol availability to reduce levels of consumption, alcohol misuse, related harm or social problems - Competing interests: Not disclosed Funding: - Centre for Public Health Excellence, National Institute for Health and Clinical Excellence	- Effectiveness; cost-effectiveness - Outcome: Alcohol consumption (consumption in the past 30 days) - Population: Adults and young people aged 10 years and above - Search: 2008 (from inception) - No. of studies included: 2 umbrella reviews (4 independent primary studies)	A quality checklist for reviews was developed. A subjective cutoff score of 9 criteria fulfilled (of a total of 14) was deemed of higher quality; only 3 broad quality scores provided	There was inconclusive evidence from 2 reviews on the impact of advertising bans on alcohol consumption. Associations were modest or non-significant One review indicated an advertising ban was likely more cost-effective than other alcohol macro-interventions. The evidence, however, was not strong *Magnitude of effect*: Unclear	*Study eligibility criteria*: Low *Identification and selection of studies:* Low *Data collection and study appraisal:* Low *Synthesis and findings*: Low Risk of bias: Low
Anderson et al., 2009;* Lancet* - Umbrella review - Effectiveness and cost-effectiveness of policies and programs to reduce the harm caused by alcohol - Competing interests: Disclosed (none reported) Funding: - Not disclosed	- Effectiveness; cost-effectiveness - Outcome: Alcohol consumption (effectiveness and cost-effectiveness) - Population: Unclear - Search: Unclear - No. of studies included: 0	None. The quality of included studies was not assessed. The strength of the evidence was based on the type of study design of included studies: 1 = more than one systematic review; 2 = one systematic review; 3 = two or more randomized controlled trials; 4 = one randomized controlled trial; 5 = observational evidence; 6 = not assessed	A comprehensive advertising ban can be cost-effective if fully enforced. Conclusions based on exposure to advertising, not on regulating the volume of advertising *Magnitude of effect*: Estimates for the cost per DALY saved ranged from 931 to 955 in 2005 international dollars	*Study eligibility criteria*: Unclear (eligibility criteria not described) *Identification and selection of studies:* Unclear (too little information provided) *Data collection and study appraisal:* High (reviews not appraised for risk of bias) *Synthesis and findings*: High (potential biases not accounted for; findings incompletely reported) Risk of bias: High
Booth et al., 2008; *Report* (University of Sheffield) - Umbrella review - The effect of industry self-regulation of alcohol advertising on alcohol consumption - Competing interests: Disclosed (none reported) Funding: - Policy Research Program, Department of Health	- Effectiveness - Outcome: Population and individual alcohol consumption, intention to consume, and substitution of alcohol - Underage drinkers, youth adult binge drinkers, harmful/heavy drinkers, low income - Search: 2000–2008 - No. of studies included: 0 review, 4 primary studies	None. Bradford Hill’s principles were used as an aid for examining causation	Inconclusive evidence that advertising bans reduce consumption, which may be attributable to differences in contextual factors. Bans may have an additive effect on other measures of control *Magnitude of effect*: Unclear	*Study eligibility criteria*: Low *Identification and selection of studies:* High (limited search; limited details on screening) *Data collection and study appraisal:* High (data extraction not described; no quality assessment) *Synthesis and findings*: Unclear (no published protocol; too little information provided) Risk of bias: High
Siegfried et al., 2014; *Cochrane Database Syst Rev* - Narrative review/meta-analysis - To evaluate the benefits, harms and costs of restricting or banning the advertising of alcohol on alcohol consumption in adults and adolescents - Competing interests: Coauthors member of WHO Working Group on Alcohol Taxation and Pricing, and WHO Expert Panel on Drug Dependence and Alcohol Problems Funding: - Medical Research Council, South Africa	- Effectiveness; cost-effectiveness - Outcome: Alcohol consumption (e.g., sales per capita, percent change in mean consumption), delayed age of initiation of alcohol use, and alcohol-related outcomes - Population: Adults of any age and adolescents (10–19 years of age) - Search: Oct 2013 or May 2014 depending on the database (from inception) - No. of studies included: 3 primary studies	Risk of bias was assessed using the *Cochrane Handbook for Systematic Reviews of Interventions*	There was a lack of robust evidence for or against recommending the implementation of alcohol advertising restrictions *Magnitude of effect*: A meta-analysis of two studies that evaluated the implementation of a ban showed an overall mean non-significant increase in beer consumption in the general population of 1.10% following the ban (95% CI –5.26, 7.47)	*Study eligibility criteria*: Low *Identification and selection of studies*: Low *Data collection and study appraisal*: Low *Synthesis and findings*: Low Risk of bias: Low
Manthey et al., 2024; *Addiction* - Narrative review - Effects of regulating alcohol advertisement, marketing, and/or sponsoring on alcohol consumption - Competing interests: Disclosed (none reported) Funding: - German Ministry of Health	- Effectiveness - Outcome: Alcohol consumption - Population: General population - Search: Oct 2022 (from inception) - No. of studies included: 11 primary studies	Risk of bias assessed using an approach which covered the four risk of bias domains outlined in the Cochrane handbook, which also structure the ROBINS-I tool: confounding, selection bias, information bias, and reporting bias	Overall, there was insufficient evidence to conclude that alcohol marketing restrictions constituted an effective tool to reduce alcohol consumption *Magnitude of effect*: N/a	*Study eligibility criteria*: Low *Identification and selection of studies*: Low *Data collection and study appraisal*: Low *Synthesis and findings*: Low Risk of bias: Low
Esser & Jernigan, 2018; *Annu Rev Public Health* - Narrative review - Policy options for regulating exposure to alcohol marketing and the use and effectiveness of these approaches - Competing interests: Disclosed (none reported) Funding: - Disclosed (none reported)	- Effectiveness, cost-effectiveness - Outcome: Alcohol consumption (specific measure not described) - Population: Not described - Search: Not described - No. of studies included: 1 umbrella review; 3 primary studies (economic evaluations)	None	Some evidence shows that complete alcohol marketing restrictions were cost-effective and associated with reducing harmful use of alcohol if well enforced *Magnitude of effect*: Unclear	*Study eligibility criteria*: Unclear (eligibility criteria not described) *Identification and selection of studies:* Unclear (too little information provided) *Data collection and study appraisal:* Unclear (too little information provided) *Synthesis and findings*: Unclear (too little information provided) Risk of bias: Unclear
Hastings et al., 2005; *J Public Health Policy* - Narrative review - What are the associations between alcohol advertising and behaviour? - Competing interests: Not disclosed Funding: - WHO	- Effectiveness - Outcome: Alcohol consumption (not described further) - Population: Young people and the general population - Search: Unclear - No. of studies included: 3 primary studies	None	Two time-series cross-sectional studies found that OECD countries with advertising bans had lower levels of alcohol consumption; one time-series cross-sectional study found that US state-level advertising bans were not associated with lower total alcohol consumption *Magnitude of effect*: Unclear	*Study eligibility criteria*: Unclear (too little information provided) *Identification and selection of studies:* Unclear (too little information provided) *Data collection and study appraisal:* High (limited data extraction table provided) *Synthesis and findings*: Unclear (too little information provided) Risk of bias: Unclear
Grube & Waiters, 2005; *Adolesc Med Clin* - Narrative review - Effects of alcohol in the media on youth - Competing interests: Not disclosed Funding: - National Institute on Alcohol Abuse and Alcoholism, National Institutes of Health	- Effectiveness - Outcome: Alcohol consumption (specific measure not described) - Population: Youth - Search: Not specified - No. of studies included: 6 primary studies	None	Some evidence suggested that advertising restrictions may be associated with decreased consumption *Magnitude of effect*: Unclear	*Study eligibility criteria*: Unclear (too little information provided) *Identification and selection of studies:* Unclear (too little information provided) *Data collection and study appraisal:* High (too little information provided) *Synthesis and findings*: High (too little information provided) Risk of bias: Unclear
**Introducing warning labels**				
Siegfried & Parry, 2019; *PLoS One* - Umbrella review - Effectiveness of policies to limit the availability of alcohol - Competing interests: Disclosed (GAPA, UNODC, WHO) Funding: - Drug Research Unit of the South African Medical Research Council	- Effectiveness; cost-effectiveness - Outcome: Alcohol consumption (not described further) - Population: General population - Search: Jan 2006–Jul 2017 - No. of studies included: 1 review (2 independent primary studies)	ROBIS; only broad scores (high, low, or uncertain) presented	Only broad conclusions provided. There was “uncertain” evidence that warning labels were associated with lower alcohol use *Magnitude of effect*: Unclear	*Study eligibility criteria*: Low *Identification and selection of studies:* Low *Data collection and study appraisal:* High (extraction process not described; included reviews’ characteristics not appropriately presented) *Synthesis and findings*: High (synthesis of results restricted to reviews at low risk of bias) Risk of bias: High
Burton et al., 2017; *Lancet* - Umbrella review/narrative rapid review - Effectiveness and cost-effectiveness of policies to reduce alcohol-related harms in England - Competing interests: Disclosed (none reported) Funding: - Review was commissioned by the Department of Health; resources were provided by Public Health England	- Effectiveness; cost-effectiveness - Outcome: Alcohol consumption - Population: Adults with some underage consumption data - Search: 2000 to 2016 - No. of studies included: 2 reviews (unclear number of independent primary studies; at least 2)	GRADE	The associations between health warning labels or restrictions on packaging with alcohol consumption were unclear *Magnitude of effect*: Unclear	*Study eligibility criteria*: Unclear (no pre-defined protocol; some inappropriate eligibility criteria) *Identification and selection of studies:* Low *Data collection and study appraisal:* High (study characteristics not provided; GRADE not designed to assess risk of bias) *Synthesis and findings*: Unclear (findings incompletely reported) Risk of bias: High
Knai et al., 2015; *Addiction* - Umbrella review - Effectiveness of alcohol labelling on reducing alcohol consumption - Competing interests: Disclosed (none reported) Funding: - Not disclosed	- Effectiveness - Outcome: Alcohol consumption (specific outcome measures not clearly specified) - Population: General population of any age group - Search: Up until Dec 2013 (unclear whether from inception) - No. of studies included: 3 reviews, 1 book (unclear number of independent primary studies; at least 4)	None. The quality of included studies was not assessed. The strength of the evidence was based on the type of study design of included studies: 1 = systematic reviews; 2 = reviews with three core criteria; i.e., evidence of comprehensive search, clear selection (inclusion/exclusion) criteria and process of quality assessment of papers reviewed; 3 = reviews not meeting the criteria of level 2	Four reviews found limited or no association between warning labels and drinking behaviour or alcohol consumption but increase awareness *Magnitude of effect:* Unclear	*Study eligibility criteria*: Low *Identification and selection of studies:* High (too little information provided) *Data collection and study appraisal:* High (limited data extraction table provided) *Synthesis and findings*: Unclear (no published protocol; limited data on synthesis methods) Risk of bias: High
Anderson et al., 2009; *Lancet* - Umbrella review - Effectiveness and cost-effectiveness of policies and programs to reduce the harm caused by alcohol - Competing interests: Disclosed (none reported) Funding: - Not disclosed	- Effectiveness; cost-effectiveness - Outcome: Unclear - Population: Unclear - Search: Unclear - No. of studies included: 1 review (3 independent primary studies)	None. The quality of included studies was not assessed. The strength of the evidence was based on the type of study design of included studies: 1 = more than one systematic review; 2 = one systematic review; 3 = 2 or more randomized controlled trials; 4 = one randomized controlled trial; 5 = observational evidence; 6 = not assessed	Mandated health warnings on alcohol products were not associated with changes in drinking behaviour, although there were associated changes in some related variables such as intention to change drinking patterns *Magnitude of effect*: Unclear	*Study eligibility criteria*: Unclear (eligibility criteria not described) *Identification and selection of studies:* Unclear (too little information provided) *Data collection and study appraisal:* High (reviews not appraised for risk of bias) *Synthesis and findings*: High (potential biases not accounted for; findings incompletely reported) Risk of bias: High
Stockwell, 2006; *Report* (Centre for Addictions Research of British Columbia, University of Victoria) - Umbrella review/narrative review - Impact of change in Canadian warning labels policy on alcohol consumption - Competing interests: Not disclosed Funding: - Health Canada	- Effectiveness - Outcome: Alcohol consumption and related behaviours - Population: General population, not further specified - Search: 1995–2004 - No. of studies included: 5 reviews, 1 book; 6 primary studies (unclear number of independent primary studies, at least 6)	None	There was little evidence of an association between warning labels and consumption *Magnitude of effect*: N/a	*Study eligibility criteria*: Unclear (too little information provided) *Identification and selection of studies:* Unclear (no information on screening process; unpublished reports not included) *Data collection and study appraisal:* High (studies not appraised for risk of bias) *Synthesis and findings*: High (potential biases not accounted for) Risk of bias: High
Clarke et al., 2021; *Health Psychol Rev* - Narrative review/meta-analysis - Impact of HWLs on selection and consumption of food, alcohol, and non-alcoholic drinks - Competing interests: Disclosed (none reported) Funding: - Wellcome Trust	- Effectiveness - Outcome: Alcohol consumption; consumption speed - Population: Children or adults - Search: Inception to Sept 2019 - No. of studies included: 0	Cochrane Risk of Bias 2.0 Tool, only broad scores (low, high, some concerns) presented	No studies included in the current review assessed amount consumed *Magnitude of effect*: N/a	*Study eligibility criteria*: Low *Identification and selection of studies:* Low *Data collection and study appraisal:* Low *Synthesis and findings*: Low Risk of bias: Low
Joyce et al., 2023; *J Addict Dis* - Narrative review - Impact of alcohol warning labels on intentions/behaviours, knowledge/awareness, perceptions, and attitudes - Competing interests: Not disclosed Funding: - CIHR; Qatar National Library; SSHRC	- Effectiveness - Outcome: alcohol consumption (drinking behaviours, and sales) - Population: general population - Search: 1979–2020 - No. of studies included: 6 primary studies	The Evidence Project Risk of Bias Tool; eight items coded yes/no: (1) cohort, (2) control or comparison group, (3) pre-post intervention data, (4) random assignment of participants to the intervention, (5) random selection of participants for assessment, (6) follow-up rate of 80% or more, (7) comparison groups equivalent on sociodemographics, (8) comparison groups equivalent at baseline on outcome measures. Only yes/no reported	Findings on attitudes/beliefs and drinking intentions/behaviour were mixed *Magnitude of effect*: Unclear	*Study eligibility criteria*: Low *Identification and selection of studies:* Low *Data collection and study appraisal:* Low *Synthesis and findings*: High (synthesis did not include all studies; potential biases not accounted) Risk of bias: Low
Hassan & Shiu, 2018; *Journal of Social Marketing* - Narrative review - Effectiveness of alcohol warning label factors in increasing acceptance of message and change in behaviour - Competing interests: Not disclosed Funding: - Not disclosed	- Effectiveness - Outcome: drinking behaviour, compliance, and attitudes towards warnings - Population: General population, including adolescents and students - Search: 2000–2015 - No. of studies included: 2 primary studies	Mixed Methods Appraisal Tool (MMAT); only global scores (*, **, ***, ****) presented	Only two studies examined consumption as an outcome; warning did not appear to significantly increase or decrease alcohol consumption *Magnitude of effect:* N/a	*Study eligibility criteria*: Low *Identification and selection of studies:* Low *Data collection and study appraisal:* Low *Synthesis and findings*: Low Risk of bias: Low
Scholes-Balog et al., 2012; *Aust N Z J Public Health* - Narrative review - Evaluate the impact of alcohol warning labels on adolescent drinking, knowledge, and behaviour - Competing interests: Not disclosed Funding: - Not disclosed	- Effectiveness - Outcome: Drinking behaviour (e.g., binge drinking) - Population: Adolescent - Search: Not reported - No. of studies included: 2 primary studies	None	Only two studies examined consumption as an outcome; labels appeared unlikely to change adolescent drinking behaviours *Magnitude of effect:* N/a	*Study eligibility criteria*: Low *Identification and selection of studies:* High (complete search terms not available for review; procedure for screening not described) *Data collection and study appraisal:* High (no risk of bias assessment; unclear how data extraction was completed) *Synthesis and findings*: High Risk of bias: High
Wilkinson et al., 2009; *Report* (National Drug Research Institute) - Narrative review - The effectiveness of alcohol warning labels - Competing interests: Not disclosed Funding: - Not disclosed	- Effectiveness - Outcome: Alcohol consumption, risky drinking - Population: General population - Search: Not reported - No. of studies included: 4 primary studies	Studies assessed for (1) strength and appropriateness of methodological design (e.g., cross-sectional vs. longitudinal data, use of matched pairs vs. unmatched controls); (2) external validity (e.g., representative/random sample, generalizability, adequate sample size, consideration of confounding and historical factors, plausibility of assumptions); and (3) internal validity (e.g., validity and reliability of measurement instruments, random allocation of subjects)	There was a very limited evidence base about the impact of alcohol warning labels on behaviour *Magnitude of effect:* N/a	S*tudy eligibility criteria*: Unclear (insufficient information reported on eligibility) *Identification and selection of studies:* Low *Data collection and study appraisal:* High (study characteristics not adequately described; risk of bias assessment not clearly reported) *Synthesis and findings*: Low Risk of bias: High
Wilkinson & Room, 2009; *Drug Alcohol Rev* - Narrative review - The effectiveness of alcohol warning labels on awareness and drinking behaviour - Competing interests: Not disclosed Funding: - Alcohol Education and Rehabilitation Foundation Ltd; Department of Human Services, State of Victoria	- Effectiveness - Outcome: Drinking behaviour - Population: General population - Search: Not reported - No. of studies included: 3 primary studies	None; included studies “critiqued in relation to methodological rigour, reliability, validity and generalisability.” Unclear how any of these components were operationalized	Most methodologically sound evaluations of alcohol warning labels were based on the US experience and found little evidence that the introduction of the warning label had an impact on drinking behaviour *Magnitude of effect:* N/a	*Study eligibility criteria*: Unclear (insufficient information reported on eligibility) *Identification and selection of studies:* Unclear (insufficient information reported on search and screening) *Data collection and study appraisal:* High (study characteristics not adequately described; no risk of bias assessment) *Synthesis and findings*: High (unclear if there were departures from pre-defined analyses; primary study bias not adequately described/assessed) Risk of bias: high

*AMSTAR* A MeaSurement Tool to Assess systematic Reviews, *GAPA* Global Alcohol Policy Alliance, *RCT* randomized controlled trial, *ROBIS* Risk Of Bias In Systematic reviews, *UNODC* United Nations Office on Drugs and Crime, *WHO* World Health Organization

### Addressing alcohol availability

#### Minimum purchasing age

We included four umbrella reviews (Anderson et al., 2009; Jackson et al., 2010; Martineau et al., 2013; Stockings et al., 2016) and one review (Wagenaar & Toomey, 2002) that examined policies that introduced or increased the minimum purchasing age. The reviews included a total of 49 independent primary studies, 48 of which were included in the review (Wagenaar & Toomey, 2002).

The most recent umbrella review (Stockings et al., 2016) which focused on young people aged 10–24 years included an older umbrella review (Jackson et al., 2010). While the most recent review of primary studies was published in 2002 and fell outside our inclusion criteria (Wagenaar & Toomey, 2002), we included it for comprehensiveness. Two umbrella reviews, assessed at low (Jackson et al., 2010) and high risk of bias (Martineau et al., 2013), concluded there was consistent evidence that a higher minimum legal drinking age was associated with a reduction in alcohol consumption, although the magnitudes of the effects were unclear. Another umbrella review that focused on young people, assessed at a high risk of bias, found inconclusive evidence that increasing the minimum legal drinking age was associated with a significant reduction of alcohol consumption (Stockings et al., 2016), while an older umbrella review, also assessed at a high risk of bias, concluded that minimum drinking age laws were associated with a reduction of youth drinking (Anderson et al., 2009).

Finally, a 2002 review of 48 primary studies, assessed at high risk of bias, found that the evidence suggested that higher legal drinking ages reduced alcohol consumption. It also concluded that the methodological quality of the studies of specific populations, such as college students, was poor, preventing any conclusions (Wagenaar & Toomey, 2002).

Although four of the five included reviews sought evidence on the cost-effectiveness of minimum purchasing age, none commented on its cost-effectiveness (Anderson et al., 2009; Jackson et al., 2010; Martineau et al., 2013; Stockings et al., 2016).

#### Days/hours of sale

We included six umbrella reviews (Anderson et al., 2009; Burton et al., 2017; Jackson et al., 2010; Martineau et al., 2013; Siegfried & Parry, 2019; Stockings et al., 2016) and six reviews (Bryden et al., 2012; Hahn et al., 2010; Kilian et al., 2023; Middleton et al., 2010; Popova et al., 2009; Sherk et al., 2018) including two recent meta-analyses (Kilian et al., 2023; Sherk et al., 2018) that examined the association between changes to the hours or days of sale and alcohol consumption. The umbrella reviews included a total of 14 independent reviews, while the six included reviews presented evidence from 27 independent primary studies.

The most recent umbrella review (Siegfried & Parry, 2019), assessed at a high risk of bias, included five reviews (Bryden et al., 2012; Hahn et al., 2010, 2012; Middleton et al., 2010; Popova et al., 2009) and concluded there was “uncertain” evidence that making changes to the hours and days of alcohol sales were associated with lower alcohol use. Three additional umbrella reviews assessed at low (Jackson et al., 2010) and high (Burton et al., 2017; Stockings et al., 2016) risks of bias similarly concluded that the evidence was limited and mixed. Two older umbrella reviews assessed at a high risk of bias concluded that restricting days or hours of sale was consistently associated with lower alcohol consumption, while increasing the days/hours of alcohol sale availability was associated with increased total alcohol consumption (Anderson et al., 2009; Martineau et al., 2013).

A 2023 meta-analysis, assessed at a low risk of bias, concluded from ten primary studies that restrictions on temporal availability (days/hours of sale) were associated with a statistically significant decrease in alcohol consumption, driven by decreases in beer and wine consumption; restriction of alcohol sales by 1 day was associated with 3.6% (95%CI − 5.1, − 2.2) lower alcohol consumption (Kilian et al., 2023). A 2018 meta-analysis, assessed at a high risk of bias, similarly concluded from six primary studies that there was an association between days and hours of sale and alcohol use with one additional day of sale (Sunday/Saturday) being associated with per capita consumption increases of 3.4% (95%CI 2.7, 4.1) for total alcohol, 5.3% (95%CI 3.2, 7.4) for beer, 2.6% (95%CI 1.8, 3.5) for wine, and 2.6% (95%CI 2.1, 3.2) for spirits (Sherk et al., 2018). Older reviews, based on few primary studies, concluded there was mixed and inconsistent evidence (Hahn et al., 2010) or limited evidence of an association between temporal availability and alcohol use (Bryden et al., 2012; Middleton et al., 2010; Popova et al., 2009).

Of seven reviews that examined cost-effectiveness (Anderson et al., 2009; Burton et al., 2017; Jackson et al., 2010; Martineau et al., 2013; Middleton et al., 2010; Siegfried & Parry, 2019; Stockings et al., 2016), only two commented on the cost-effectiveness of restricting days and hours of sales (Jackson et al., 2010; Middleton et al., 2010). One review highlighted one study which indicated that reducing licensed hours of sale was relatively low cost (Jackson et al., 2010), while another review included one modelling study which concluded that restricting alcohol sales for a 24-h period over the weekend was very cost-effective (Middleton et al., 2010).

#### Alcohol outlet density

We included five umbrella reviews (Anderson et al., 2009; Burton et al., 2017; Jackson et al., 2010; Martineau et al., 2013; Stockings et al., 2016) and five reviews (Bryden et al., 2012; Campbell et al., 2009; Gmel et al., 2016; Popova et al., 2009; Sherk et al., 2018), including one meta-analysis (Sherk et al., 2018), that examined the association between alcohol outlet density and alcohol consumption. The reviews included a total of 37 independent primary studies.

A 2010 umbrella review, rated at a low risk of bias, concluded based on two reviews and 11 additional primary studies that there was a positive association between outlet density and alcohol consumption in adults and young people across different study designs and measures of consumption (Jackson et al., 2010). Conversely, a 2017 umbrella review, rated at a high risk of bias, concluded from seven reviews that there was limited and mixed evidence about the association between outlet density and alcohol consumption (Burton et al., 2017). A further three older umbrella reviews, all rated at a high risk of bias, generally concluded that there was a positive association between alcohol outlet density and alcohol consumption (Anderson et al., 2009; Martineau et al., 2013; Stockings et al., 2016). None of the umbrella reviews commented on the magnitude of the effects.

The most recent review, rated at a low risk of bias, found an association between outlet density and alcohol consumption in Canada and the United States (US); three Canadian studies reported outlet elasticities of alcohol between 0.07 and 0.19, while one US study found an increase in outlet density of one standard deviation was associated with a 7% increase in consumption for men and 11% for women (Sherk et al., 2018).

Older reviews rated at low (Bryden et al., 2012; Campbell et al., 2009) and high (Gmel et al., 2016; Popova et al., 2009) risks of bias reached different conclusions. Some found consistent evidence of a positive association between outlet density and alcohol consumption (Campbell et al., 2009; Popova et al., 2009), while others found mixed evidence of the effectiveness of reducing alcohol outlet density to reduce consumption (Bryden et al., 2012; Gmel et al., 2016). Based on eight primary studies, one review reported mean outlet elasticities (on- and off-premises aggregated, 0.27; on-premises only, 0.25; off-premises only, 2.46) (Campbell et al., 2009).

Of the six reviews that examined cost-effectiveness (Anderson et al., 2009; Burton et al., 2017; Campbell et al., 2009; Jackson et al., 2010; Martineau et al., 2013; Stockings et al., 2016), three commented on the cost-effectiveness of limiting alcohol density (Anderson et al., 2009; Burton et al., 2017; Campbell et al., 2009). One umbrella review (Burton et al., 2017), from one review assessed at high risk of bias (Anderson et al., 2009), indicated that the effect of reducing access to retail outlets for specified periods of the week had the potential to be very cost-effective. One review assessed at a low risk of bias failed to identify a single study but pointed out that even in the absence of published data on program implementation costs and other costs related to restricting outlet density, it should be expected that the cost of restricting access to alcohol by limiting the number of alcohol outlets was likely to be small relative to the societal cost of excessive alcohol consumption (Campbell et al., 2009).

#### Retail privatization/monopolization

We identified two umbrella reviews assessed at a high risk of bias (Anderson et al., 2009; Martineau et al., 2013) and one narrative systematic review assessed at a low risk of bias (Hahn et al., 2012) that examined the retail privatization or monopolization of alcohol sales. The reviews included a total of 16 independent primary studies, 15 of which were included in one review (Hahn et al., 2012). Overall, the evidence suggested that privatization was associated with increases in excessive drinking and alcohol sales, with government monopolization having the opposite association. The systematic review included 13 studies with quasi-experimental or cross-sectional designs and reported a median increase in per capita sales of 44% (IQR 5, 123) across study periods, with some inconsistency between primary studies that may be attributed to methodological differences (e.g., drink classifications) (Hahn et al., 2012). For non-privatized beverages, privatization was associated with a median decrease in sales of non-privatized alcoholic beverages of 2% (IQR 0 to 7). These decreases were not enough to offset the overall increase from privatized beverages.

One review identified one modelling study that estimated the cost of privatization versus government monopolies in Canada (Hahn et al., 2012). Modelling the alcohol-attributable burden and associated costs (direct healthcare costs (acute care and psychiatric hospitalizations costs, outpatient and inpatient specialized treatment costs, ambulatory care’s physician fees, family physician visits costs, prescription drug costs), indirect costs (productivity losses due to premature mortality, and short- and long-term disability), and the direct costs of criminality), it was estimated that the privatization of provincial and territorial alcohol monopolies would increase costs by 12% (Can$1.6 billion) (Popova et al., 2012).

### Addressing alcohol marketing

#### Self-regulation

We identified two umbrella reviews, both assessed at a high risk of bias (Anderson et al., 2009; Booth et al., 2008), that examined the association between marketing self-regulation and alcohol consumption. An umbrella review failed to identify any evidence for or against self-regulation of alcohol advertising (Booth et al., 2008), while another umbrella review concluded, based on one primary study, that self-regulation did not prevent types of marketing that can affect young people (Anderson et al., 2009). This conclusion, however, was not supported by the evidence presented.

#### Advertising from government authorities to reduce alcohol use

We identified four umbrella reviews, all assessed at a high risk of bias (Anderson et al., 2009; Booth et al., 2008; Martineau et al., 2013; Stockings et al., 2016), and two reviews assessed at a low risk of bias (Bryden et al., 2012; Young et al., 2018) that examined the association between advertising from government authorities and alcohol use. The reviews included a total of 20 independent primary studies, 13 of which were included in one review (Young et al., 2018). The two reviews concluded that there was little evidence that government-funded counter-advertising mass media campaigns reduced alcohol use. Three of the four umbrella reviews reached a similar conclusion (Anderson et al., 2009; Booth et al., 2008; Stockings et al., 2016), while one pointed to “low quality” evidence from two meta-analyses that found mass media campaigns were associated with reduced alcohol use (Martineau et al., 2013).

#### Regulating the volume of advertising from alcohol manufacturers

We included nine umbrella reviews (Anderson et al., 2009; Booth et al., 2008; Burton et al., 2017; Jackson et al., 2010; Knai et al., 2015; Martineau et al., 2013; Petticrew et al., 2017; Siegfried & Parry, 2019; Stockings et al., 2016) and five reviews (Esser & Jernigan, 2018; Grube & Waiters, 2005; Hastings et al., 2005; Manthey et al., 2024; Siegfried et al., 2014) that examined regulating the volume of advertising from alcohol manufacturers. The reviews included a total of 20 independent primary studies. All but two (Jackson et al., 2010; Siegfried et al., 2014) were assessed at high or unclear risks of bias. Conclusions from umbrella reviews and reviews were based on few reviews and primary studies, respectively. Umbrella reviews and reviews generally found that there was a lack of direct evidence for or against recommending the implementation of alcohol advertising restrictions (Booth et al., 2008; Burton et al., 2017; Hastings et al., 2005; Jackson et al., 2010; Knai et al., 2015; Martineau et al., 2013; Petticrew et al., 2017; Siegfried & Parry, 2019; Siegfried et al., 2014; Stockings et al., 2016). A 2005 review, assessed at an unclear risk of bias, concluded from six primary studies that some evidence suggested that advertising restrictions may have decreased consumption (Grube & Waiters, 2005).

Two umbrella reviews and a review, rated at high and unclear risks of bias, pointed out that modelling studies indicated that alcohol marketing restrictions were cost-effective (Burton et al., 2017; Esser & Jernigan, 2018; Stockings et al., 2016). This conclusion, however, was based on a few modelling studies that assumed advertising restrictions were effective at reducing alcohol consumption (Chisholm et al., 2004; Cobiac et al., 2009; Hollingworth et al., 2006; Holm et al., 2014; Meier et al., 2009).

#### Introducing warning labels

We included five umbrella reviews (Anderson et al., 2009; Burton et al., 2017; Knai et al., 2015; Siegfried & Parry, 2019; Stockwell, 2006) and six reviews (Clarke et al., 2020; Hassan & Shiu, 2018; Joyce et al., 2023; Scholes-Balog et al., 2012; Wilkinson & Room, 2009; Wilkinson et al., 2009) that examined the association between warning labels and alcohol consumption. Umbrella reviews, all assessed at a high risk of bias, were in agreement that the evidence was sparse and did not allow for definitive conclusions. Reviews, of which three of six were assessed at a low risk of bias (Clarke et al., 2020; Hassan & Shiu, 2018; Joyce et al., 2023), reached similar conclusions. The reviews included a total of 11 independent primary studies.

### Risk of bias assessment

We found that reviews most often failed to adequately assess the quality/risk of bias of the evidence. Fifteen reviews did not assess the risk of bias of included studies (Anderson et al., 2009; Booth et al., 2008; Esser & Jernigan, 2018; Gmel et al., 2016; Grube & Waiters, 2005; Hastings et al., 2005; Knai et al., 2015; Petticrew et al., 2017; Popova et al., 2009; Scholes-Balog et al., 2012; Sherk et al., 2018; Stockings et al., 2016; Stockwell, 2006; Wagenaar & Toomey, 2002; Wilkinson & Room, 2009); five did not clearly report the assessment for each included study (Campbell et al., 2009; Hahn et al., 2010, 2012; Middleton et al., 2010; Wilkinson et al., 2009); nine reviews only reported broad or yes/no scores (Bryden et al., 2012; Clarke et al., 2021; Hassan & Shiu, 2018; Jackson et al., 2010; Joyce et al., 2023; Kilian et al., 2023; Martineau et al., 2013; Siegfried & Parry, 2019; Young et al., 2018); and one used a tool not designed to assess risk of bias (Burton et al., 2017).

Funding and competing interests were clearly disclosed in all but nine reviews (Anderson et al., 2009; Bryden et al., 2012; Grube & Waiters, 2005; Hahn et al., 2012; Hastings et al., 2005; Jackson et al., 2010; Middleton et al., 2010; Popova et al., 2009; Wagenaar & Toomey, 2002). However, only one review reported funding and potential competing interest of included studies (Siegfried et al., 2014), while two reported that included studies were funded by the alcohol industry (Joyce et al., 2023; Martineau et al., 2013), and one excluded studies with alcohol industry funding (Burton et al., 2017).

## Discussion

Existing reviews provide consistent evidence that addressing alcohol availability (introducing or increasing minimum purchasing age, restrictions on temporal availability (days/hours of sale), decreasing outlet density, government monopolization) was associated with lower alcohol use. To elaborate, (1) overall, the evidence from reviews, although somewhat outdated, indicated that introducing or increasing minimum purchasing age was associated with reduced alcohol consumption. The magnitudes of effects were, however, unclear. (2) Although most included reviews concluded that the evidence suggesting an association between changes in days and hours of alcohol sale was limited and mixed, two recent meta-analyses (one of which assessed at a low risk of bias) concluded that restrictions on temporal availability (days/hours of sale) were associated with a statistically significant decrease in alcohol consumption, with effect sizes likely to be policy meaningful (Kilian et al., 2023; Sherk et al., 2018). (3) Overall, the included reviews pointed to a positive association between alcohol outlet density and alcohol consumption. The most recent review, reporting on four primary studies conducted in the US and Canada, found effect sizes that were likely policy meaningful (Sherk et al., 2018). (4) Although somewhat dated, the evidence from reviews suggested that privatization was associated with increases in excessive drinking and alcohol sales, while government monopolization was associated with decreased alcohol use, with changes in sales due to privatization/monopolization likely to be policy meaningful (Anderson et al., 2009; Hahn et al., 2012; Martineau et al., 2013). The findings that changes in outlet density and temporal availability were associated with alcohol use add support to an association between privatization/monopolization and alcohol use as they are two plausible causal pathways.

There was a general lack of evidence on the associations between alcohol marketing (marketing self-regulation, advertising from government authorities, regulating the volume of advertising from alcohol manufacturers, and introducing warning labels) and alcohol consumption, which precludes any conclusions about these regulations. It is important to note that our findings should not be interpreted as evidence that alcohol marketing does not affect alcohol use. Rather, we conclude that the evidence from reviews was too limited to make any conclusive statements about the impact of alcohol marketing on alcohol use. It is also important to note that complete marketing bans have seldom been implemented, making their evaluation difficult (i.e., much of the literature has focused on the effectiveness of partial restrictions and not comprehensive bans) (Burton et al., 2017). In the tobacco control context, there is substantial evidence that comprehensive bans are effective, while partial marketing bans have little or no effect (US National Cancer Institute & World Health Organization, 2016).

Although a number of reviews aimed to examine both the effectiveness and cost-effectiveness of population-level policies to reduce alcohol use, evidence actually assessing cost-effectiveness was extremely scarce (Anderson et al., 2009; Burton et al., 2017; Campbell et al., 2009; Esser & Jernigan, 2018; Hahn et al., 2012; Jackson et al., 2010; Martineau et al., 2013; Middleton et al., 2010; Siegfried & Parry, 2019; Siegfried et al., 2014; Stockings et al., 2016). This is likely due, at least in part, to the relatively low cost of implementation and enforcement of population-level policies such as minimum purchasing age, and restrictions on outlet density, temporal availability, and the marketing of alcohol (Campbell et al., 2009).

### Limitations

Given the strength of the evidence linking alcohol use to ill health, we focused solely on reviews that examined the effectiveness and cost-effectiveness of population-level policies to reduce alcohol use, resulting in the exclusion of several reviews that examined other outcomes such as violence, motor vehicle fatalities, injury, hospitalizations, and crime. Consequently, our findings are not necessarily generalizable to broader alcohol-related harms and thus likely underestimate the overall benefit of some of the policies examined. Similarly, our focus on alcohol use led us to exclude reviews that examined more indirect outcomes. For example, it has been argued that assessing the evidence on alcohol marketing and marketing restrictions requires analyses beyond evaluations of population-based advertising restrictions which employ a broader systems perspective, as marketing aims to influence not just consumption, but also awareness, attitudes, and social norms (Petticrew et al., 2017). Similarly, alcohol health warning labels may affect, in addition to alcohol use, knowledge or awareness of risks or harms from alcohol, perceptions, attention, and intentions to change drinking behaviour (Joyce et al., 2023).

Our umbrella review excluded an important population-level policy domain, and price and tax strategies. A recent umbrella review identified 30 reviews and concluded there was substantial evidence indicating that increased alcohol prices and taxes led to a reduction in overall alcohol consumption, with the degree of price sensitivity differing among various types of beverages (Guindon et al., 2022). Included reviews generally found that increased taxes and prices were associated with reductions in heavy episodic drinking and heavy drinking, though the extent of these associations was often unclear (Guindon et al., 2022). The umbrella review identified one review that examined non-tax pricing strategies, which concluded that price-based alcohol policy interventions, such as minimum unit pricing, were likely to reduce alcohol consumption (Boniface et al., 2017).

Although we are confident about the direction of the associations we examined, many reviews failed to clearly report, or even comment, on the magnitude of the associations. As a result, for some policies, we had difficulties going beyond the direction of effects.

Umbrella reviews may be based on a similar pool of reviews, while reviews may be based on a similar pool of primary studies. Our mapping of reviews and primary studies, for each policy domain, revealed fairly limited overlap. This lack of overlap in many policy domains is perplexing. Umbrella reviews and reviews’ failure to provide a clear list of excluded studies with reasons for exclusions makes it difficult to explain.

For several policy domains, the number of umbrella reviews we identified relative to reviews surprised us, as did the scarcity of recent reviews (as opposed to recent umbrella reviews). For one policy domain, minimum purchasing age, we were unable to identify a single review of primary studies published since 2005 (our inclusion criterion). For comprehensiveness, we included a review published in 2002 which included 48 primary studies published between 1975 and 1998. Future research should prioritize reviews over umbrella reviews and focus on policy domains with more limited recent evidence.

## Conclusion

The Government of Ontario began expanding privatized alcohol sales in 2015 with initial plans for further expansions in 2026 (Giesbrecht & Myran, 2024; Schwartz et al., 2023). In a surprising May 24, 2024, announcement, the Ontario government indicated the expansion would begin in August 2024. From November 1, 2024, all eligible grocery and big-box stores were able to sell beer, cider, wine, and ready-to-drink beverages, including in large pack sizes (Government of Ontario, 2024). This expansion may result in a nearly 300% increase in the number of alcohol outlets in Ontario (Giesbrecht & Myran, 2024). The evidence presented in this review suggests that the expansion of outlets will likely result in an increase in alcohol sales. Additionally, this expansion may put downward pressure on prices, which would result in increased alcohol consumption (Guindon et al., 2022).

Much of the evidence reviewed focused on average effects (with the exception of age) with limited discussion of the potential of policies to have heterogeneous effects between groups (e.g., sex/gender, race/ethnicity, socioeconomic status). One recent review specifically sought evidence on differences in policy effects across groups and found little to no research (Kilian et al., 2023). Recent and forthcoming changes in alcohol policies in Canada present a unique research opportunity to examine the real-world impact of policies while paying particular attention to group differences.

## Contributions to knowledge

What does this study add to existing knowledge?

- This umbrella review provides an up-to-date inventory of reviews with a risk of bias assessment of all included reviews that have examined the effectiveness and cost-effectiveness of population-level policies to reduce alcohol use.

What are the key implications for public health interventions, practice, or policy?

- Addressing alcohol availability (introducing or increasing minimum purchasing age, restrictions on temporal availability, decreasing outlet density, government monopolization) can reduce alcohol use.
- There is a lack of evidence on the associations between alcohol marketing (marketing self-regulation, advertising from government authorities, regulating the volume of advertising from alcohol manufacturers, and introducing warning labels) and alcohol consumption, which precludes any conclusions about these regulations.

## Supplementary Information

Below is the link to the electronic supplementary material.

Supplementary file1 (PDF 1525 KB)

Supplementary file2 (XLSX 46 KB)

Supplementary file3 (XLSX 36 KB)

## Data Availability

All data generated or analyzed during this study are included in this article (and its supplementary files).
