# Supplementary material for: The effectiveness and cost-effectiveness of population-level policies to reduce alcohol use: A systematic umbrella review
Source: Can J Public Health. 2025 Apr 3;116(6):951–84. doi: 10.17269/s41997-025-01013-9 (PMC12753591; doi:10.17269/s41997-025-01013-9)
Supplement: Supplementary file 1 — Supplementary file1 (PDF 1525 KB) [file 41997_2025_1013_MOESM1_ESM.pdf]

Appendix 1. Search Strategy

Limits consistent between all databases: published since January 2005, English and French

1) Cochrane ((Alcohol **AND** harm) OR (alcohol* AND (abuse OR misuse OR addict* OR consum* OR dependen* OR drink* OR drinking behaviour*)) OR alcohol drinking pattern* OR alcohol-related disorder*) **AND** (marketing OR social marketing OR advertis* OR packag* OR product packag* OR warning* OR health warning label* OR health warnings OR warning label OR self-regulation OR alcohol trading hours OR alcohol outlet OR alcohol sales OR alcohol availability OR closing time OR outlet density OR hours of sale OR drinking hour* OR (operating OR open* or close* hour*) OR privatiz* OR monopol* OR minimum age OR drinking age OR legal age OR purchase age)

Limited to topic area: "tobacco, drugs alcohol", "alcohol"

2) EconLit ((Alcohol **AND** harm) OR (alcohol* AND (abuse OR misuse OR addict* OR consum* OR dependen* OR drink* OR drinking behaviour*)) OR alcohol drinking pattern* OR alcohol-related disorder*) **AND** (marketing OR social marketing OR advertis* OR packag* OR product packag* OR warning* OR health warning label* OR health warnings OR warning label OR self-regulation OR alcohol trading hours OR alcohol outlet OR alcohol sales OR alcohol availability OR closing time OR outlet density OR hours of sale OR drinking hour* OR (operating OR open* or close* hour*) OR privatiz* OR monopol* OR minimum age OR drinking age OR legal age OR purchase age) **AND** Review

Limits: last 15 years, reviews

3) HealthEvidence (www.healthevidence.org) alcohol **AND** (market* OR availab*)

Limits: last 15 years, topic area limited to addiction/substance use and alcohol abuse/use

4) Health Systems Evidence (www.healthsystemsevidence.org) alcohol* (searched for overviews of systematic reviews, systematic reviews of effects and systematic reviews addressing other questions)

5) Medline ((Alcohol **AND** harm) OR (alcohol* AND (abuse OR misuse OR addict* OR consum* OR dependen* OR drink* OR drinking behaviour*)) OR alcohol drinking pattern* OR alcohol-related disorder*) **AND** (marketing OR social marketing OR advertis* OR packag* OR product packag* OR warning* OR health warning label* OR health warnings OR warning label OR self-regulation OR alcohol trading hours OR alcohol outlet OR alcohol sales OR alcohol availability OR closing time OR outlet density OR hours of sale OR drinking hour* OR (operating OR open* or close* hour*) OR privatiz* OR monopol* OR minimum age OR drinking age OR legal age OR purchase age)

Limits: reviews, published within the past 15 years

6) PubMed (policy **AND** (economic evaluation or cost-effective* or economic analys*) **AND** ((Alcohol and harm) or "alcohol abuse" or "alcohol use" or "alcoholic" or "alcoholism" or "alcohol misuse" or "alcohol addiction" or "alcohol consumption" or "alcohol dependence" or "alcohol use disorder"))

Filters: From 2018/1/1 to 2020/10/30 (to update the search conducted in a previous version of this rapid synthesis)

7) PsychInfo ((Alcohol **AND** harm) OR (alcohol* AND (abuse OR misuse OR addict* OR consum* OR dependen* OR drink* OR drinking behaviour*)) OR alcohol drinking pattern* OR alcohol-related disorder*) **AND** (marketing OR social marketing OR advertis* OR packag* OR product packag* OR warning* OR health warning label* OR health warnings OR warning label OR self-regulation OR alcohol trading hours OR alcohol outlet OR alcohol sales OR alcohol availability OR closing time OR outlet

density OR hours of sale OR drinking hour* OR (operating OR open* or close* hour*) OR privatiz* OR monopol* OR minimum age OR drinking age OR legal age OR purchase age)

Limits: reviews, published within the past 15 years

8) Social Systems Evidence (www.socialsystemsevidence.org) alcohol **AND** (market* OR availab*)

Limits: last 15 years

PRIOR Checklist

(Gates M, Gates A, Pieper D, et al. Reporting guideline for overviews of reviews of healthcare interventions: development of the PRIOR statement. ***BMJ*** **2022;378:e070849. doi:10.1136/bmj-2022-070849.)**

**Section** Topic	**#**	**Item**	**Location reported**
**TITLE**			
Title	1	Identify the report as an overview of reviews.	Title page
**ABSTRACT**			
Abstract	2	Provide a comprehensive and accurate summary of the purpose, methods, and results of the overview of reviews.	P2
**INTRODUCTION**			
Rationale	3	Describe the rationale for conducting the overview of reviews in the context of existing knowledge.	P5
Objectives	4	Provide an explicit statement of the objective(s) or question(s) addressed by the overview of reviews.	P5
**METHODS**			
Eligibility criteria	5a	Specify the inclusion and exclusion criteria for the overview of reviews. If supplemental primary studies were included, this should be stated, with a rationale.	P6
	5b	Specify the definition of 'systematic review' as used in the inclusion criteria for the overview of reviews.	P6
Information sources	6	Specify all databases, registers, websites, organizations, reference lists, and other sources searched or consulted to identify systematic reviews and supplemental primary studies (if included). Specify the date when each source was last searched or consulted.	P6
Search strategy	7	Present the full search strategies for all databases, registers and websites, such that they could be reproduced. Describe any search filters and limits applied.	Appendix 1
Selection process	8a	Describe the methods used to decide whether a systematic review or supplemental primary study (if included) met the inclusion criteria of the overview of reviews.	P6,7
	8b	Describe how overlap in the populations, interventions, comparators, and/or outcomes of systematic reviews was identified and managed during study selection.	P6,7
Data collection process	9a	Describe the methods used to collect data from reports.	P6,7
	9b	If applicable, describe the methods used to identify and manage primary study overlap at the level of the comparison and outcome during data collection. For each outcome, specify the method used to illustrate and/or quantify the degree of primary study overlap across systematic reviews.	Appendix 4
	9c	If applicable, specify the methods used to manage discrepant data across systematic reviews during data collection.	P6,7 Tables 1-2
Data items	10	List and define all variables and outcomes for which data were sought. Describe any assumptions made and/or measures taken to identify and clarify missing or unclear information.	P6,7
Risk of bias assessment	11a	Describe the methods used to *assess* risk of bias or methodological quality of the included systematic reviews.	P6,7
	11b	Describe the methods used to *collect* data on (from the systematic reviews) and/or *assess* the risk of bias of the primary studies included in the systematic reviews. Provide a justification for instances where flawed, incomplete, or missing assessments are identified but not re-assessed.	P6,7
	11c	Describe the methods used to *assess* the risk of bias of supplemental primary studies (if included).	N/A
Synthesis methods	12a	Describe the methods used to summarize or synthesize results and provide a rationale for the choice(s).	P6,7
	12b	Describe any methods used to explore possible causes of heterogeneity among results.	P7
	12c	Describe any sensitivity analyses conducted to assess the robustness of the synthesized results.	P7
Reporting bias assessment	13	Describe the methods used to *collect* data on (from the systematic reviews) and/or *assess* the risk of bias due to missing results in a summary or synthesis (arising from reporting biases at the levels of the systematic reviews, primary studies, and supplemental primary studies, if included).	P7
Certainty assessment	14	Describe the methods used to *collect* data on (from the systematic reviews) and/or *assess* certainty (or confidence) in the body of evidence for an outcome.	P6,7
**RESULTS**			
Systematic review and supplemental primary study selection	15a	Describe the results of the search and selection process, including the number of records screened, assessed for eligibility, and included in the overview of reviews, ideally with a flow diagram.	P7
	15b	Provide a list of studies that might appear to meet the inclusion criteria, but were excluded, with the main reason for exclusion.	P7, Fig 1 Appendix 3

**Section** Topic	#	**Item**	**Location reported**
Characteristics of systematic reviews and supplemental primary studies	16	Cite each included systematic review and supplemental primary study (if included) and present its characteristics.	P7-11 Tables 1,2
Primary study overlap	17	Describe the extent of primary study overlap across the included systematic reviews.	Appendix 3
Risk of bias in systematic reviews, primary studies, and supplemental primary studies	18a	Present assessments of risk of bias or methodological quality for each included systematic review.	Tables 1,2 Appendix 4
	18b	Present assessments (*collected* from systematic reviews or *assessed* anew) of the risk of bias of the primary studies included in the systematic reviews.	Tables 1,2
	18c	Present assessments of the risk of bias of supplemental primary studies (if included).	N/A
Summary or synthesis of results	19a	For all outcomes, summarize the evidence from the systematic reviews and supplemental primary studies (if included). If meta-analyses were done, present for each the summary estimate and its precision and measures of statistical heterogeneity. If comparing groups, describe the direction of the effect.	P7-11 Tables 1,2
	19b	If meta-analyses were done, present results of all investigations of possible causes of heterogeneity.	P7-11 Tables 1,2
	19c	If meta-analyses were done, present results of all sensitivity analyses conducted to assess the robustness of synthesized results.	P7-11 Tables 1,2
Reporting biases	20	Present assessments (*collected* from systematic reviews and/or *assessed* anew) of the risk of bias due to missing primary studies, analyses, or results in a summary or synthesis (arising from reporting biases at the levels of the systematic reviews, primary studies, and supplemental primary studies, if included) for each summary or synthesis assessed.	P7-11 Tables 1,2
Certainty of evidence	21	Present assessments (*collected* or *assessed* anew) of certainty (or confidence) in the body of evidence for each outcome.	P7-11 Tables 1,2
**DISCUSSION**			
Discussion	22a	Summarize the main findings, including any discrepancies in findings across the included systematic reviews and supplemental primary studies (if included).	P11,12
	22b	Provide a general interpretation of the results in the context of other evidence.	P11,12
	22c	Discuss any limitations of the evidence from systematic reviews, their primary studies, and supplemental primary studies (if included) included in the overview of reviews. Discuss any limitations of the overview of reviews methods used.	P11,12
	22d	Discuss implications for practice, policy, and future research (both systematic reviews and primary research). Consider the relevance of the findings to the end users of the overview of reviews, e.g., healthcare providers, policymakers, patients, among others.	P11,12
**OTHER INFORMATION**			
Registration and protocol	23a	Provide registration information for the overview of reviews, including register name and registration number, or state that the overview of reviews was not registered.	P6
	23b	Indicate where the overview of reviews protocol can be accessed, or state that a protocol was not prepared.	P6
	23c	Describe and explain any amendments to information provided at registration or in the protocol. Indicate the stage of the overview of reviews at which amendments were made.	P6
Support	24	Describe sources of financial or non-financial support for the overview of reviews, and the role of the funders or sponsors in the overview of reviews.	Title page
Competing interests	25	Declare any competing interests of the overview of reviews' authors.	Title page
Author information	26a	Provide contact information for the corresponding author.	Title page
	26b	Describe the contributions of individual authors and identify the guarantor of the overview of reviews.	Title page
Availability of data and other materials	27	Report which of the following are available, where they can be found, and under which conditions they may be accessed: template data collection forms; data collected from included systematic reviews and supplemental primary studies; analytic code; any other materials used in the overview of reviews.	Title page

Appendix 3. Excluded studies, by reason of exclusion

Wrong outcome (n=17)

Baldwin R, Miller PG, Coomber K, Patafio B, Scott D. A systematic narrative review of the effects of alcohol supply reduction policies on children and adolescents. *International Journal of Drug Policy*. 2022 Mar 1;101:103581.

Berdzuli N, Ferreira-Borges C, Gual A, Rehm J. Alcohol control policy in Europe: Overview and exemplary countries. *International journal of environmental research and public health*. 2020 Nov;17(21):8162.

Cusens B, Shepherd J. Prevention of alcohol-related assault and injury. *British Journal of Hospital Medicine* (2005). 2005 Jun;66(6):346-8.

Fitterer JL, Nelson TA, Stockwell T. A review of existing studies reporting the negative effects of alcohol access and positive effects of alcohol control policies on interpersonal violence. *Frontiers in Public Health*. 2015 Nov 16;3:170308.

Fitzgerald N, Angus K, Emslie C, Shipton D, Bauld L. Gender differences in the impact of population-level alcohol policy interventions: evidence synthesis of systematic reviews. *Addiction*. 2016 Oct;111(10):1735-47.

Harding FM, Hingson RW, Klitzner M, Mosher JF, Brown J, Vincent RM, Dahl E, Cannon CL. Underage drinking: a review of trends and prevention strategies. *American journal of preventive medicine*. 2016 Oct 1;51(4):S148-57.

Kokole D, Anderson P, Jané-Llopis E. Nature and potential impact of alcohol health warning labels: A scoping review. Nutrients. 2021 Aug 31;13(9):3065.

Nelson JP. Alcohol marketing, adolescent drinking and publication bias in longitudinal studies: a critical survey using meta-analysis. *Journal of Economic Surveys.* 2011 Apr;25(2):191-232.

Nelson JP, McNall AD. Alcohol prices, taxes, and alcohol-related harms: A critical review of natural experiments in alcohol policy for nine countries. *Health Policy.* 2016 Mar 1;120(3):264-72.

Nepal S, Kypri K, Pursey K, Attia J, Chikritzhs T, Miller P. Effectiveness of lockouts in reducing alcohol-related harm: systematic review. *Drug and alcohol review*. 2018 May;37(4):527-36.

Pena S, Sierralta P, Norambuena P, Leyton F, Pemjean A, Roman F. Alcohol policy in Chile: a systematic review of policy developments and evaluations. *Addiction*. 2021 Mar;116(3):438-56.

Sanchez-Ramirez DC, Voaklander D. The impact of policies regulating alcohol trading hours and days on specific alcohol-related harms: a systematic review. *Injury prevention*. 2018 Feb 1;24(1):94-100.

Taylor N, Miller P, Coomber K, Mayshak R, Zahnow R, Patafio B, Burn M, Ferris J. A mapping review of evaluations of alcohol policy restrictions targeting alcohol-related harm in night-time entertainment precincts. *International Journal of Drug Policy.* 2018 Dec 1;62:1-3.

Wagenaar AC, Tobler AL, Komro KA. Effects of alcohol tax and price policies on morbidity and mortality: a systematic review. *American journal of public health*. 2010 Nov;100(11):2270-8.

Wilkinson C, Livingston M, Room R. Impacts of changes to trading hours of liquor licences on alcohol-related harm: a systematic review 2005–2015. *Public health research & practice.* 2016 Sep 30.

Wilson IM, Graham K, Taft A. Alcohol interventions, alcohol policy and intimate partner violence: a systematic review. *BMC public health.* 2014 Dec;14:1-1.

Yadav RP, Kobayashi M. A systematic review: effectiveness of mass media campaigns for reducing alcohol-impaired driving and alcohol-related crashes. *BMC public health*. 2015 Dec;15:1-7.

Wrong study design (n=13)

Anderson P. Is it time to ban alcohol advertising?. *Clinical Medicine*. 2009 Apr 4;9(2):121.

Babor TF, Caetano R. Evidence-based alcohol policy in the Americas: strengths, weaknesses, and future challenges. *Revista Panamericana de Salud Pública*. 2005 Nov;18(4-5):327-37.

Boggs MM, Praveen Durgampudi MB. The impact of exposure to alcohol advertisements on adolescents: A literature review. *International Public Health Journal*. 2017;9(1):13.

Casswell S. Profits or people? The informative case of alcohol marketing. *The New Zealand Medical Journal (Online)*. 2014 Nov 28;127(1406):87.

Nelson JP, McNall AD. What happens to drinking when alcohol policy changes? A review of five natural experiments for alcohol taxes, prices, and availability. *The European Journal of Health Economics.* 2017 May;18(4):417-34.

Nemtsov A, Neufeld M, Rehm J. Are trends in alcohol consumption and cause-specific mortality in Russia between 1990 and 2017 the result of alcohol policy measures?. *Journal of studies on alcohol and drugs.* 2019 Sep;80(5):489-98.

Neufeld M, Bobrova A, Davletov K, Štelemėkas M, Stoppel R, Ferreira-Borges C, Breda J, Rehm J. Alcohol control policies in Former Soviet Union countries: a narrative review of three decades of policy changes and their apparent effects. *Drug and alcohol review*. 2021 Mar;40(3):350-67.

Neufeld M, Rehm J. Alcohol consumption and mortality in Russia since 2000: are there any changes following the alcohol policy changes starting in 2006?. *Alcohol and alcoholism.* 2013 Mar 1;48(2):222-30.

Neufeld M, Rehm J. Effectiveness of policy changes to reduce harm from unrecorded alcohol in Russia between 2005 and now. *International Journal of Drug Policy*. 2018 Jan 1;51:1-9.

Sargent JD, Babor TF. The relationship between exposure to alcohol marketing and underage drinking is causal. *Journal of Studies on Alcohol and Drugs*, Supplement. 2020 Mar(s19):113-24.

Sharma A, Sinha K, Vandenberg B. Pricing as a means of controlling alcohol consumption. *British medical bulletin.* 2017 Sep 1;123(1):149-58.

Stockwell T, Sherk A, Norström T, Angus C, Ramstedt M, Andréasson S, Chikritzhs T, Gripenberg J, Holder H, Holmes J, Mäkelä P. Estimating the public health impact of disbanding a government alcohol monopoly: application of new methods to the case of Sweden. *BMC public health*. 2018 Dec;18(1):1-6.

Xu X, Chaloupka FJ. The effects of prices on alcohol use and its consequences. *Alcohol Research & Health*. 2011;34(2):236.

Wrong/unclear intervention (n=7)

Anderson P, Kokole D, Llopis EJ. Production, consumption, and potential public health impact of low- and no-alcohol products: results of a scoping review. *Nutrients*. 2021 Sep 10;13(9):3153.

Brennan I, Moore SC, Byrne E, Murphy S. Interventions for disorder and severe intoxication in and around licensed premises, 1989–2009. *Addiction*. 2011 Apr;106(4):706-13.

Lachenmeier DW, Taylor BJ, Rehm J. Alcohol under the radar: do we have policy options regarding unrecorded alcohol?. *International Journal of Drug Policy*. 2011 Mar 1;22(2):153-60.

Li Q, Babor TF, Zeigler D, Xuan Z, Morisky D, Hovell MF, Nelson TF, Shen W, Li B. Health promotion interventions and policies addressing excessive alcohol use: a systematic review of national and global evidence as a guide to health-care reform in C hina. *Addiction*. 2015 Jan;110:68-78.

Marteau TM, Jebb SA, Lewis HB, Wei Y, Higgins JP, Ogilvie D. *Portion, package or tableware size for changing selection and consumption of food, alcohol and tobacco*. Cochrane database of systematic reviews. 2015(9).

Scott S, Muirhead C, Shucksmith J, Tyrrell R, Kaner E. Does industry-driven alcohol marketing influence adolescent drinking behaviour? A systematic review. *Alcohol and alcoholism.* 2017 Jan 1;52(1):84-94.

Stautz K, Brown KG, King SE, Shemilt I, Marteau TM. Immediate effects of alcohol marketing communications and media portrayals on consumption and cognition: a systematic review and meta-analysis of experimental studies. *BMC Public Health.* 2016 Dec;16:1-8.

Wrong publication type (n=4)

Casswell S. Current status of alcohol marketing policy—an urgent challenge for global governance. *Addiction*. 2012 Mar;107(3):478-85.

Cukier S, Wettlaufer A, Jackson K, Minozzi S, Bartholow BD, Stoolmiller ML, Sargent JD, Cochrane Public Health Group. Impact of exposure to alcohol marketing and subsequent drinking patterns among youth and young adults. *Cochrane database of systematic reviews*. 1996 Sep 1;2018(8).

Pärna K. Alcohol consumption and alcohol policy in Estonia 2000–2017 in the context of Baltic and Nordic countries. *Drug and Alcohol Review*. 2020 Nov;39(7):797-804.

Wechsler H, Nelson TF. Will increasing alcohol availability by lowering the minimum legal drinking age decrease drinking and related consequences among youths?. *American journal of public health.* 2010 Jun;100(6):986-92.

Wrong population (n=3)

Grigoriev P, Bobrova A. Alcohol control policies and mortality trends in Belarus. *Drug and alcohol review*. 2020 Nov;39(7):805-17.

Jiang H, Xiang X, Hao W, Room R, Zhang X, Wang X. Measuring and preventing alcohol use and related harm among young people in Asian countries: a thematic review. *Global health research and policy*. 2018 Dec;3:1-4.

Medina-Mora ME, Monteiro M, Rafful C, Samano I. Comprehensive analysis of alcohol policies in the Latin America and the Caribbean. *Drug and Alcohol Review.* 2021 Mar;40(3):385-401.

Appendix 4. List of studies included in each 'umbrella reviews of reviews' and 'reviews'

Addressing alcohol availability: minimum purchasing age

Umbrella reviews, included (4)

Stockings, Hall et al., 2016
- Number of reviews: 1 (Jackson, Johnson et al., 2010)

Martineau, Tyner et al., 2013
- Number of reviews: 2 (Wagenaar, Toomey, 2000, 2002)

Jackson, Johnson et al., 2010
- Number of reviews: 1 (Wagenaar, Toomey, 2002)
- Number of primary studies: 1 (DiNardo, Lemieux, 2001)

Anderson, Chisholm, Fuhr, 2009
- Number of reviews: 1 (Wagenaar, Toomey, 2000)

Reviews, included (1)

Wagenaar, Toomey, 2002
- Number of primary studies: 48 (Arndt, 1988; Barsby and Marshall, 1977; Bellows, 1980; Bessmer, 1984; Coate and Grossman, 1987, 1988; Colon, 1980; Davis and Reynolds, 1990; Dee, 1999; Douglass and Millar, 1979; Engs and Hanson, 1988; George et al., 1989; Gonzalez, 1989, 1990a, b; Gordon and Minor, 1992; Grossman et al., 1994; Hingson et al., 1983; Hoadley et al., 1984; Hughes and Dodder, 1986; Hughes and Dodder, 1992; Johnson et al., 1992; Laixuthai, 1994; Laixuthai and Chaloupka, 1993; Lillis et al., 1987; Lonnstrom, 1985; Lotterhos et al., 1988; Maisto and Rachal, 1980; McCornac and Filante, 1984; McFadden and Wechsler, 1979; Mooney and Gramling, 1993; Mooney et al., 1992; O'Malley and Wagenaar, 1991; Ornstein, 1984; Perkins and Berkowitz, 1989; Rooney and Schwartz, 1977; Schweitzer et al., 1983; Smart, 1977; Smart and Finley, 1976; Smart and Schmidt, 1975; Smith et al., 1984; Vingilis and Smart, 1981; Wagenaar, 1982a; Wagenaar, 1982b; Wilkinson, 1987; Williams and Lillis, 1986, 1988; Yu and Shacket, 1998)

Umbrella reviews, all (4)

1. Anderson P, Chisholm D, Fuhr DC. Effectiveness and cost-effectiveness of policies and programmes to reduce the harm caused by alcohol. The Lancet. 2009;373(9682):2234-2246. doi:10.1016/S0140-6736(09)60744-3
2. Jackson R, Johnson M, Campbell F, et al. Interventions on Control of Alcohol Price, Promotion and Availability for Prevention of Alcohol Use Disorders in Adults and Young People. Sheffield: School of Health and Related Research, University of Sheffield;2010.
3. Martineau F, Tyner E, Lorenc T, Petticrew M, Lock K. Population-level interventions to reduce alcohol-related harm: an overview of systematic reviews. Prev Med. Oct 2013;57(4):278-96. doi:10.1016/j.ypmed.2013.06.019
4. Stockings E, Hall WD, Lynskey M, et al. Prevention, early intervention, harm reduction, and treatment of substance use in young people. Lancet Psychiatry. Mar 2016;3(3):280-96. doi:10.1016/s2215-0366(16)00002-x

Reviews, all (2)

1. Wagenaar AC, Toomey TL. Alcohol Policy: Gaps between Legislative Action and Current Research. Contemporary Drug Problems. 2000;27(4):681-733.
2. Wagenaar AC, Toomey TL. Effects of minimum drinking age laws: review and analyses of the literature from 1960 to 2000. J Stud Alcohol, Supplement. 2002;(s14):206-25. doi:10.15288/jsas.2002.s14.206

Primary studies, all (49)

1. Arndt SK. Adolescent alcohol consumption and a changing legal drinking age. H.S.D. Indiana University; 1988.
2. Barsby SL, Marshall GL. Short-term consumption effects of a lower minimum alcohol-purchasing age. J Stud Alcohol. Sep 1977;38(9):1665-79. doi:10.15288/jsa.1977.38.1665
3. Bellows DC. The Effects of Lowering the Drinking Age in Nebraska: A Quasi-Experimental Time-Series Analysis. Ph.D. University of Nebraska-Lincoln; 1980.
4. Bessmer MA. The relationship of self-reported problems and drinking among college students. Southern Illinois University at Carbondale; 1984.
5. Coate D, Grossman M. Change in Alcoholic Beverage Prices and Legal Drinking Ages: Effects on Youth Alcohol Use and Motor Vehicle Mortality. Alcohol Health and Research World. 1987;12(1):22.
6. Coate D, Grossman M. Effects of Alcoholic Beverage Prices and Legal Drinking Ages on Youth Alcohol Use. The Journal of Law & Economics. 1988;31(1):145-171.
7. Colon I. Alcohol Control Policies and Their Relation to Alcohol Consumption and Alcoholism. Ph.D. The Heller School for Social Policy and Management, Brandeis University; 1980.
8. Davis JE, Reynolds NC. Alcohol use among college students: responses to raising the purchase age. J Am Coll Health. May 1990;38(6):263-9. doi:10.1080/07448481.1990.9936198
9. Dee TS. State alcohol policies, teen drinking and traffic fatalities. Journal of Public Economics. 1999/05/01/ 1999;72(2):289-315. doi:10.1016/S0047-2727(98)00093-0
10. DiNardo J, Lemieux T. Alcohol, marijuana, and American youth: the unintended consequences of government regulation. Journal of health economics. 2001;20(6):991-1010. doi:10.1016/S0167-6296(01)00102-3
11. Douglass RL, Millar CW. Alcohol availability and alcohol-related casualties in Michigan 1968-1976. Curr Alcohol. 1979 1979;6:303-317.
12. Engs RC, Hanson DJ. University students' drinking patterns and problems: examining the effects of raising the purchase age. Public Health Rep. Nov-Dec 1988;103(6):667-73.
13. George WH, Crowe LC, Abwender D, Skinner JB. Effects of Raising the Drinking Age to 21 Years in New York State on Self-Reported Consumption by College Students1. Journal of Applied Social Psychology. 1989/06/01 1989;19(8):623-635. doi:10.1111/j.1559-1816.1989.tb00343.x
14. Gonzalez GM. Effects of raising the drinking age among college students in Florida. College Student Journal. 1989;23(1):62-75.
15. Gonzalez GM. Effects of raising the drinking age and related campus initiatives on student alcohol consumption and alcohol-related problems. Journal of College Student Development. 1990;31(2):181–183.
16. Gonzalez GM. Effects of Drinking Age on Reduced Consumption of Alcohol Reported by College Students: 1981–1986. Journal of Drug Issues. 1990/01/01 1990;20(1):67-73. doi:10.1177/002204269002000104
17. Gordon RA, Minor SW. Attitudes toward a change in the legal drinking age: Reactance versus compliance. Journal of College Student Development. 1992;33(2):171–176.
18. Grossman M, Chaloupka FJ, Saffer H, Laixuthai A. Effects of Alcohol Price Policy on Youth: A Summary of Economic Research. Journal of Research on Adolescence. 1994/04/01 1994;4(2):347-364. doi:10.1207/s15327795jra0402_9

19. Hingson RW, Scotch N, Mangione T, et al. Impact of legislation raising the legal drinking age in Massachusetts from 18 to 20. American journal of public health. Feb 1983;73(2):163-70. doi:10.2105/ajph.73.2.163
20. Hoadley JF, Fuchs BC, Holder HD. The effect of alcohol beverage restrictions on consumption: a 25-year longitudinal analysis. Am J Drug Alcohol Abuse. 1984;10(3):375-401. doi:10.3109/00952998409001478
21. Hughes SP, Dodder RA. Raising the Legal Minimum Drinking Age: Short-Term Effects with College Student Samples. Journal of Drug Issues. 1986/10/01 1986;16(4):609-620. doi:10.1177/002204268601600408
22. Hughes SP, Dodder RA. Changing the legal minimum drinking age: results of a longitudinal study. J Stud Alcohol. Nov 1992;53(6):568-75. doi:10.15288/jsa.1992.53.568
23. Johnson JA, Oksanen EH, Veall MR, Fretz D. Short-Run and Long-Run Elasticities for Canadian Consumption of Alcoholic Beverages: an Error-Correction Mechanism/Cointegration Approach. The Review of Economics and Statistics. 1992;74(1):64-74. doi:10.2307/2109543
24. Laixuthai A. Youth alcohol use and abuse: An examination of public policy and laws on alcohol, marijuana, and drunk driving. Ph.D. University of Illinois at Chicago; 1994.
25. Laixuthai A, Chaloupka FJ. Youth Alcohol Use and Public Policy. Contemporary Economic Policy. 1993/10/01 1993;11(4):70-81. doi:10.1111/j.1465-7287.1993.tb00402.x
26. Lillis RP, Williams TP, Williford WR. Impact of the 19-year- old drinking age in New York. In: Holder HD, ed. Advances in Substance Abuse: Behavioral and Biological Research, Supplement 1: Control Issues in Alcohol Abuse Prevention: Strategies for States and Communities. JAI Press; 1987:133-146.
27. Lonnstrom D. Impact of Changes in the New York State Minimum Drinking Age Law upon the College Campus. Rensselaer Polytechnic Institute; 1985.
28. Lotterhos JF, Glover ED, Holbert D, Barnes RC. Intentionality of college students regarding North Carolina's 21-year drinking age law. The International journal of the addictions. Jun 1988;23(6):629-47. doi:10.3109/10826088809039225
29. Maisto SA, Rachal JV. Indications of the relationship among adolescent drinking practices, related behaviors, and drinking-age laws. In: H W, ed. Minimum-Drinking-Age Laws: An Evaluation. 1980:155-176.
30. McCornac DC, Filante RW. The demand for distilled spirits: an empirical investigation. J Stud Alcohol. Mar 1984;45(2):176-8. doi:10.15288/jsa.1984.45.176
31. McFadden M, Wechsler H. Minimum drinking age laws and teenage drinking. Psychiatric opinion. 1979;16(3):22-23, 26-28.
32. Mooney LA, Gramling R. The Differential Effects of the Minimum Drinking Age Law. Sociological Inquiry. 1993;63(3):330-338. doi:10.1111/j.1475-682X.1993.tb00313.x
33. Mooney LA, Gramling R, Forsyth C. Legal drinking age and alcohol consumption. Deviant Behavior. 1992/01/01 1992;13(1):59-71. doi:10.1080/01639625.1992.9967898
34. O'Malley PM, Wagenaar AC. Effects of minimum drinking age laws on alcohol use, related behaviors and traffic crash involvement among American youth: 1976-1987. J Stud Alcohol. Sep 1991;52(5):478-91. doi:10.15288/jsa.1991.52.478
35. Ornstein SI. A survey of findings on the economic and regulatory determinants of the demand for alcoholic beverages. Subst Alcohol Actions Misuse. 1984;5(1):39-44.
36. Perkins HW, Berkowitz AD. Stability and Contradiction in College Students' Drinking Following a Drinking-Age Law Change. Journal of Alcohol and Drug Education. 1989;35(1):60-77.
37. Rooney JF, Schwartz SM. The effect of minimum drinking age laws upon adolescent alcohol use and problems. Contemp Drug Probs. 1977;6:569.

38. Schweitzer SO, Intriligator MD, Salehi H. Alcoholism: an econometric model of its causes, its effects, and its control. In: Grant M, Williams A, Plant M, eds. Economics and Alcohol. 1st Edition ed. Routledge; 1983:107-27.

39. Smart RG. Changes in alcoholic beverage sales after reductions in the legal drinking age. Am J Drug Alcohol Abuse. 1977;4(1):101-8. doi:10.3109/00952997709002751

40. Smart RG, Finley J. Changes in drinking age and per capita beer consumption in ten Canadian provinces. Addict Dis. 1976;2(3):393-402.

41. Smart RG, Schmidt W. Drinking and problems from drinking after a reduction in the minimum drinking age. Br J Addict Alcohol Other Drugs. Dec 1975;70(4):347-58. doi:10.1111/j.1360-0443.1975.tb00048.x

42. Smith RA, Hingson RW, Morelock S, et al. Legislation raising the legal drinking age in Massachusetts from 18 to 20: effect on 16 and 17 year-olds. J Stud Alcohol. Nov 1984;45(6):534-9. doi:10.15288/jsa.1984.45.534

43. Vingilis E, Smart RG. Effects of raising the legal drinking age in Ontario. British journal of addiction. Dec 1981;76(4):415-24. doi:10.1111/j.1360-0443.1981.tb03240.x

44. Wagenaar AC. Aggregate beer and wine consumption; effects of changes in the minimum legal drinking age and a mandatory beverage container deposit law in Michigan. J Stud Alcohol. May 1982;43(5):469-87. doi:10.15288/jsa.1982.43.469

45. Wagenaar AC. Public Policy Effects on Alochol Consumption in Maine and New Hampshire: 1970-1980. Contemporary Drug Problems. 1982 1982;11(1):3-20. 20.

46. Wilkinson JT. Reducing Drunken Driving: Which Policies Are Most Effective? Southern Economic Journal. 1987;54(2):322-334. doi:10.2307/1059317

47. Williams TP, Lillis RP. Changes in alcohol consumption by 18-year-olds following an increase in New York State's purchase age to 19. J Stud Alcohol. Jul 1986;47(4):290-6. doi:10.15288/jsa.1986.47.290

48. Williams TP, Lillis RP. Long-term changes in reported alcohol purchasing and consumption following an increase in New York State's purchase age to 19. British journal of addiction. Feb 1988;83(2):209-17. doi:10.1111/j.1360-0443.1988.tb03983.x

49. Yu J, Shacket RW. Long-term change in underage drinking and impaired driving after the establishment of drinking age laws in New York State. Alcoholism, clinical and experimental research. Oct 1998;22(7):1443-9. doi:10.1111/j.1530-0277.1998.tb03933.x

Addressing alcohol availability: days/hours of sale

Umbrella reviews, included (6)

Siegfried, Parry, 2019
- Number of reviews: 5 (Bryden et al., 2012; Hahn et al., 2010, 2012; Middleton et al., 2010; Popova et al., 2009)

Burton, Henn et al., 2017
- Number of reviews: 5 (Anderson, Chisholm, Fuhr, 2009; Hahn et al., 2010; Holmes et al., 2014; Middleton et al., 2010; Organisation for Economic Co-operation and Development, 2015)
- Number of primary studies: 1 (Kypri, McElduff, Miller, 2014)

Stockings, Hall et al., 2016
- Number of reviews: 2 (Babor et al., 2010; Jackson et al., 2010; Martineau et al., 2013)

Martineau, Tyner et al., 2013
- Number of reviews: 4 (Bryden et al., 2012; Hahn et al., 2010; Middleton et al., 2010; Popova et al., 2009)

Jackson, Johnson et al., 2010
- Number of reviews: 2 (d'Abbs, Togni 2000, Mäkelä, Rossow, Tryggvesson 2002)
- Number of primary studies: 6 (UK Department for Culture, Media and Sport, 2008, Hoadley, Fuchs, Holder, 1984, Hogan et al., 2006, Hough, Hunter, 2008, Norstrom, Skog, 2005, Norström, Skog, 2003)

Anderson, Chisholm, Fuhr, 2009
- Number of reviews: 2 (Anderson, Baumberg, 2006; Babor et al., 2003)

Reviews, included (6)

Kilian, Lemp et al., 2023
- Number of primary studies: 10 (Carpenter, Eisenberg, 2009, Grönqvist, Niknami, 2014, Hough, Hunter 2008, Kolosnitsyna, Sitdikov, Khorkina 2014, Martin Bassols, Vall Castello, 2018, Nelson, 2008, Norstrom, Skog 2005, Rehm et al., 2022, Stehr 2007, Yörük 2014)

Sherk, Stockwell et al., 2018
- Number of primary studies: 7 (Carpenter, Eisenberg 2009, Grönqvist, Niknami 2014, Kolosnitsyna, Sitdikov, Khorkina, 2014, Norstrom, Skog 2005, Norström, Skog 2003, Stehr, 2007, Yörük 2014)

Bryden, Roberts et al., 2012
- Number of primary studies: 3 (Douglas, 1998; Gray et al., 2000; McLaughlin, Harrison-Stewart, 1992)

Hahn, Kuzara et al., 2010
- Number of primary studies: 4 (Bruce, 1980; Knight, Wilson, 1980; Vingilis et al., 2005; Williams, 1972)

Middleton, Hahn et al., 2010
- Number of primary studies: 8 (Chisholm et al, 2004; Knight, Wilson, 1980; McMillan, Hanson, Lapham, 2007; Norlund, 1985; Norstrom, Skog, 2005; Norström, Skog, 2003; Olsson and Wikstrom, 1982; Stehr, 2007)

Popova, Giesbrecht et al., 2009
- Number of primary studies: 3 (Chikritzhs, Stockwell, 2007; Norstrom, Skog 2005, Vingilis et al. 2005)

Umbrella reviews, all (6)

1. Anderson P, Chisholm D, Fuhr DC. Effectiveness and cost-effectiveness of policies and programmes to reduce the harm caused by alcohol. The Lancet. 2009;373(9682):2234-2246. doi:10.1016/S0140-6736(09)60744-3

2. Burton R, Henn C, Lavoie D, et al. A rapid evidence review of the effectiveness and cost-effectiveness of alcohol control policies: an English perspective. Lancet. Apr 15 2017;389(10078):1558-1580. doi:10.1016/S0140-6736(16)32420-5
3. Jackson R, Johnson M, Campbell F, et al. Interventions on Control of Alcohol Price, Promotion and Availability for Prevention of Alcohol Use Disorders in Adults and Young People. 2010.
4. Martineau F, Tyner E, Lorenc T, Petticrew M, Lock K. Population-level interventions to reduce alcohol-related harm: an overview of systematic reviews. Prev Med. Oct 2013;57(4):278-96. doi:10.1016/j.ypmed.2013.06.019
5. Siegfried N, Parry C. Do alcohol control policies work? An umbrella review and quality assessment of systematic reviews of alcohol control interventions (2006 - 2017). PLoS One. 2019;14(4):e0214865. doi:10.1371/journal.pone.0214865
6. Stockings E, Hall WD, Lynskey M, et al. Prevention, early intervention, harm reduction, and treatment of substance use in young people. Lancet Psychiatry. Mar 2016;3(3):280-96. doi:10.1016/s2215-0366(16)00002-x

Reviews, all (13)

1. Anderson P, Baumberg B. Alcohol in Europe. A public health perspective. A report for the European Commission. 2006.
2. Babor TF, Caetano R, Casswell S, et al. Alcohol: No Ordinary Commodity: Research and Public Policy. First ed. Oxford University Press; 2003. https://academic-oup-com.libaccess.lib.mcmaster.ca/book/5789
3. Bryden A, Roberts B, McKee M, Petticrew M. A systematic review of the influence on alcohol use of community level availability and marketing of alcohol. Health Place. Mar 2012;18(2):349-57. doi:10.1016/j.healthplace.2011.11.003
4. d'Abbs P, Togni S. Liquor licensing and community action in regional and remote Australia: a review of recent initiatives. Aust N Z J Public Health. Feb 2000;24(1):45-53. doi:10.1111/j.1467-842x.2000.tb00722.x
5. Hahn RA, Kuzara JL, Elder R, et al. Effectiveness of policies restricting hours of alcohol sales in preventing excessive alcohol consumption and related harms. Am J Prev Med. Dec 2010;39(6):590-604. doi:10.1016/j.amepre.2010.09.016
6. Hahn RA, Middleton JC, Elder R, et al. Effects of alcohol retail privatization on excessive alcohol consumption and related harms: a community guide systematic review. Am J Prev Med. Apr 2012;42(4):418-27. doi:10.1016/j.amepre.2012.01.002
7. Holmes J, Guo Y, Maheswaran R, Nicholls J, Meier PS, Brennan A. The impact of spatial and temporal availability of alcohol on its consumption and related harms: a critical review in the context of UK licensing policies. Drug Alcohol Rev. Sep 2014;33(5):515-25. doi:10.1111/dar.12191
8. Kilian C, Lemp JM, Llamosas-Falcon L, et al. Reducing alcohol use through alcohol control policies in the general population and population subgroups: a systematic review and meta-analysis. EClinicalMedicine. May 2023;59:101996. doi:10.1016/j.eclinm.2023.101996
9. Mäkelä P, Rossow I, Tryggvesson K. Who drinks more and less when policies change? The evidence from 50 years of Nordic studies. In: Room R, ed. The Effect of Nordic Alcohol Policies What happens to drinking and harm when alcohol controls change? NAD PUBLICATION No 42. Nordic Council for Alcohol and Drug Research (NAD); 2002:13-41.
10. Middleton JC, Hahn RA, Kuzara JL, et al. Effectiveness of policies maintaining or restricting days of alcohol sales on excessive alcohol consumption and related harms. Am J Prev Med. Dec 2010;39(6):575-89. doi:10.1016/j.amepre.2010.09.015

11. Organisation for Economic Co-operation and Development. Tackling Harmful Alcohol Use: Economics and Public Health Policy. 2015.

12. Popova S, Giesbrecht N, Bekmuradov D, Patra J. Hours and days of sale and density of alcohol outlets: impacts on alcohol consumption and damage: a systematic review. Alcohol Alcohol. Sep-Oct 2009;44(5):500-16. doi:10.1093/alcalc/agp054

13. Sherk A, Stockwell T, Chikritzhs T, et al. Alcohol Consumption and the Physical Availability of Take-Away Alcohol: Systematic Reviews and Meta-Analyses of the Days and Hours of Sale and Outlet Density. J Stud Alcohol Drugs. Jan 2018;79(1):58-67.

Primary studies, all (27)

1. Bruce D. Changes in Scottish drinking habits and behaviour following the extension of permitted evening opening hours. Health Bull. May 1980;38(3):133-7.

2. Carpenter CS, Eisenberg D. Effects of Sunday sales restrictions on overall and day-specific alcohol consumption: evidence from Canada. J Stud Alcohol Drugs. Jan 2009;70(1):126-33. doi:10.15288/jsad.2009.70.126

3. Chikritzhs T, Stockwell T. The impact of later trading hours for hotels (public houses) on breath alcohol levels of apprehended impaired drivers. Addiction. Oct 2007;102(10):1609-17. doi:10.1111/j.1360-0443.2007.01981.x

4. Chisholm D, Rehm J, Van Ommeren M, Monteiro M. Reducing the global burden of hazardous alcohol use: a comparative cost-effectiveness analysis. J Stud Alcohol. Nov 2004;65(6):782-93. doi:10.15288/jsa.2004.65.782

5. UK Department for Culture, Media and Sport. Evaluation of the impact of the Licensing Act 2003. 2008. https://webarchive.nationalarchives.gov.uk/ukgwa/20100512144753/http:/www.culture.gov.uk/images/publications/Licensingevaluation.pdf

6. Douglas M. Restriction of the hours of sale of alcohol in a small community: a beneficial impact. Aust N Z J Public Health. Oct 1998;22(6):714-9. doi:10.1111/j.1467-842x.1998.tb01476.x

7. Gray D, Saggers S, Atkinson D, Sputore B, Bourbon D. Beating the grog: an evaluation of the Tennant Creek liquor licensing restrictions. Aust N Z J Public Health. Feb 2000;24(1):39-44. doi:10.1111/j.1467-842x.2000.tb00721.x

8. Grönqvist H, Niknami S. Alcohol availability and crime: Lessons from liberalized weekend sales restrictions. Journal of Urban Economics. 2014/05/01/ 2014;81:77-84. doi:https://doi.org/10.1016/j.jue.2014.03.001

9. Hoadley JF, Fuchs BC, Holder HD. The Effect of Alcohol Beverage Restrictions on Consumption: A 25-Year Longitudinal Analysis. The American Journal of Drug and Alcohol Abuse. 1984;10(3):375-401. doi:10.3109/00952998409001478

10. Hogan E, Boffa J, Rosewarne C, Bell S, Chee DA. What price do we pay to prevent alcohol-related harms in Aboriginal communities? The Alice Springs trial of liquor licensing restrictions. Drug Alcohol Rev. May 2006;25(3):207-12. doi:10.1080/09595230600644665

11. Hough M, Hunter G. The 2003 Licensing Act's impact on crime and disorder: An evaluation. Criminology & Criminal Justice. 2008/08/01 2008;8(3):239-260. doi:10.1177/1748895808092428

12. Knight IB, Wilson P. Scottish Licensing Laws: a survey carried out on behalf of the Scottish Home and Health Department. 1980.

13. Kolosnitsyna M, Sitdikov M, Khorkina N. Availability restrictions and alcohol consumption: A case of restricted hours of alcohol sales in Russian regions. International Journal of Alcohol and Drug Research. 09/08 2014;3(3):193 – 201. doi:10.7895/ijadr.v3i3.154

14. Kypri K, McElduff P, Miller P. Restrictions in pub closing times and lockouts in Newcastle, Australia five years on. Drug Alcohol Rev. May 2014;33(3):323-6. doi:10.1111/dar.12123

15. Martin Bassols N, Vall Castello J. Bar opening hours, alcohol consumption and workplace accidents. Labour Economics. 2018/08/01/ 2018;53:172-181. doi:https://doi.org/10.1016/j.labeco.2018.04.011

16. McLaughlin KL, Harrison-Stewart AJ. The Effect of a Temporary Period of Relaxed Licensing Laws on the Alcohol Consumption of Young Male Drinkers. International Journal of the Addictions. 1992/01/01 1992;27(4):409-423. doi:10.3109/10826089209068750

17. McMillan GP, Hanson TE, Lapham SC. Geographic variability in alcohol-related crashes in response to legalized Sunday packaged alcohol sales in New Mexico. Accid Anal Prev. Mar 2007;39(2):252-7. doi:10.1016/j.aap.2006.07.012

18. Nelson JP. How Similar are Youth and Adult Alcohol Behaviors? Panel Results for Excise Taxes and Outlet Density. Atlantic Economic Journal. 2008/03/01 2008;36(1):89-104. doi:10.1007/s11293-007-9106-6

19. Norlund S. Effects of Saturday closing of wine and spirits shops in Norway, Oslo. 1985.

20. Norström T, Skog O-J. Saturday opening of alcohol retail shops in Sweden: an impact analysis. Journal of Studies on Alcohol. 2003;64(3):393-401. doi:10.15288/jsa.2003.64.393

21. Norstrom T, Skog O-J. Saturday opening of alcohol retail shops in Sweden: an experiment in two phases. Addiction. Jun 2005;100(6):767-76. doi:10.1111/j.1360-0443.2005.01068.x

22. Olsson O, Wikstrom P-OH. Effects of the experimental Saturday closing of liquor retail stores in Sweden. Contemp Drug Probl. 1982;11:325-353.

23. Rehm J, Tran A, Gobina I, et al. Do alcohol control policies have the predicted effects on consumption? An analysis of the Baltic countries and Poland 2000-2020. Drug Alcohol Depend. Dec 1 2022;241:109682. doi:10.1016/j.drugalcdep.2022.109682

24. Stehr M. The Effect of Sunday Sales Bans and Excise Taxes on Drinking and Cross—Border Shopping for Alcoholic Beverages. National Tax Journal. 2007;60(1):85-105. doi:10.17310/ntj.2007.1.05

25. Vingilis E, McLeod AI, Seeley J, Mann RE, Beirness D, Compton CP. Road safety impact of extended drinking hours in Ontario. Accid Anal Prev. May 2005;37(3):549-56. doi:10.1016/j.aap.2004.05.006

26. Williams EA. Changes in Trading Hours:Ten O'Clock Closing and Consumption of Alcohol in Victoria. Economic Record. 1972/03/01 1972;48(1):123-127. doi:10.1111/j.1475-4932.1972.tb01541.x

27. Yörük BK. Legalization of Sunday alcohol sales and alcohol consumption in the United States. Addiction. Jan 2014;109(1):55-61. doi:10.1111/add.12358

Addressing alcohol availability: alcohol outlet density

Umbrella reviews, included (5)

Burton, Henn et al., 2017

- Number of reviews: 7 (Campbell et al. 2009, Gmel, Holmes, Studer, 2016, Holmes et al. 2014, Livingston, Chikritzhs, Room, 2007, Middleton et al. 2010, Organisation for Economic Co-operation and Development 2015, Popova et al. 2009)

Stockings, Hall et al., 2016

- Number of reviews: 1 (Babor, Caetano et al., 2003)

Martineau, Tyner et al., 2013

- Number of reviews: 1 (Jackson, Johnson et al., 2010)

Jackson, Johnson et al., 2010

- Number of reviews: 2 (Her et al. 1999, Mäkelä, Rossow, Tryggvesson 2002)
- Number of primary studies: 11 (Gruenewald, Johnson, Treno 2002, Huckle et al. 2008, Kuntsche, Kuendig, Gmel 2008, Kuntsche, Kuendig 2005, Kypri et al. 2008, Livingston, Laslett, Dietze 2008, Pollack et al. 2005, Schonlau et al. 2008, Scribner, Cohen and Fisher 2000, Weitzman, Nelson, Wechsler 2003, Xie, Mann, Smart 2000)

Anderson, Chisholm, Fuhr, 2009

- Number of reviews: 1 (Livingston, Chikritzhs, Room, 2007)
- Number of primary studies: 1 (Huckle et al. 2008)

Reviews, included (5)

Sherk, Stockwell et al., 2018

- Number of primary studies: 4 (Brenner et al. 2015, Stockwell et al. 2009, Trolldal 2005, Xie, Mann, Smart 2000)

Gmel, Holmes, Studer, 2016

- Number of primary studies: 10 (Ahern et al. 2013, Connor et al. 2011, Cooper et al. 2013, Gruenewald, Remer, LaScala 2014, Halonen et al. 2014, Nordlund 2010, Picone et al. 2010, Shimotsu et al. 2013, Stockwell et al. 2009, Theall et al. 2011)

Bryden, Roberts et al., 2012

- Number of primary studies: 13 (Chen, Grube, Gruenewald 2010, Gruenewald, Johnson, Treno 2002, Huckle et al. 2008, Kuntsche, Kuendig, Gmel 2008, Kwate, Meyer 2009, Livingston, Laslett, Dietze 2008, Pollack et al. 2005, Rootman and Oakey 1973, Scribner, Cohen, Fisher 2000, Tobler, Komro and Maldonado-Molina 2009, Truong, Sturm 2009, van Oers and Garretsen 1993, Weitzman, Nelson, Wechsler 2003)

Campbell, Hahn, et al., 2009

- Number of primary studies: 8 (Blake, Nied 1997, Blose, Holder 1987, Gruenewald, Ponicki, Holder 1993, Hoadley, Fuchs, Holder 1984, Mäkelä 2002, McCornac, Filante 1984, Wagenaar, Langley 1995, Xie, Mann and Smart 2000)

Popova, Giesbrecht et al., 2009

- Number of primary studies: 11 (Gruenewald, Johnson,Treno 2002, Huckle et al. 2008, Kuntsche, Kuendig 2005, Kypri et al. 2008, Livingston, Laslett, Dietze 2008, Pollack et al. 2005, Schonlau et al. 2008, Scribner, Cohen, Fisher 2000, Scribner et al. 2008, Truong, Sturm 2009, Weitzman, Nelson, Wechsler 2003)

Umbrella reviews, all (5)

1. Anderson P, Chisholm D, Fuhr DC. Effectiveness and cost-effectiveness of policies and programmes to reduce the harm caused by alcohol. The Lancet. 2009;373(9682):2234-2246. doi:10.1016/S0140-6736(09)60744-3

2. Burton R, Henn C, Lavoie D, et al. A rapid evidence review of the effectiveness and cost-effectiveness of alcohol control policies: an English perspective. Lancet. Apr 15 2017;389(10078):1558-1580. doi:10.1016/S0140-6736(16)32420-5
3. Jackson R, Johnson M, Campbell F, et al. Interventions on Control of Alcohol Price, Promotion and Availability for Prevention of Alcohol Use Disorders in Adults and Young People. 2010.
4. Martineau F, Tyner E, Lorenc T, Petticrew M, Lock K. Population-level interventions to reduce alcohol-related harm: an overview of systematic reviews. Prev Med. Oct 2013;57(4):278-96. doi:10.1016/j.ypmed.2013.06.019
5. Stockings E, Hall WD, Lynskey M, et al. Prevention, early intervention, harm reduction, and treatment of substance use in young people. Lancet Psychiatry. Mar 2016;3(3):280-96. doi:10.1016/s2215-0366(16)00002-x

Reviews, all (10)

1. Babor TF, Caetano R, Casswell S, et al. Alcohol: No Ordinary Commodity: Research and Public Policy. Second ed. Oxford University Press; 2010.
2. Bryden A, Roberts B, McKee M, Petticrew M. A systematic review of the influence on alcohol use of community level availability and marketing of alcohol. Health Place. Mar 2012;18(2):349-57. doi:10.1016/j.healthplace.2011.11.003
3. Campbell CA, Hahn RA, Elder R, et al. The effectiveness of limiting alcohol outlet density as a means of reducing excessive alcohol consumption and alcohol-related harms. Am J Prev Med. Dec 2009;37(6):556-69. doi:10.1016/j.amepre.2009.09.028
4. Gmel G, Holmes J, Studer J. Are alcohol outlet densities strongly associated with alcohol-related outcomes? A critical review of recent evidence. Drug Alcohol Rev. Jan 2016;35(1):40-54. doi:10.1111/dar.12304
5. Her M, Giesbrecht N, Room R, Rehm J. Privatizing alcohol sales and alcohol consumption: evidence and implications. Addiction. Aug 1999;94(8):1125-39. doi:10.1046/j.1360-0443.1999.94811253.x
6. Livingston M, Chikritzhs T, Room R. Changing the density of alcohol outlets to reduce alcohol-related problems. Drug Alcohol Rev. Sep 2007;26(5):557-66. doi:10.1080/09595230701499191
7. Mäkelä P, Rossow I, Tryggvesson K. Who drinks more and less when policies change? The evidence from 50 years of Nordic studies. In: Room R, ed. The Effect of Nordic Alcohol Policies What happens to drinking and harm when alcohol controls change? NAD PUBLICATION No 42. Nordic Council for Alcohol and Drug Research (NAD); 2002:13-41.
8. Organisation for Economic Co-operation and Development. Tackling Harmful Alcohol Use: Economics and Public Health Policy. 2015.
9. Popova S, Giesbrecht N, Bekmuradov D, Patra J. Hours and days of sale and density of alcohol outlets: impacts on alcohol consumption and damage: a systematic review. Alcohol Alcohol. Sep-Oct 2009;44(5):500-16. doi:10.1093/alcalc/agp054
10. Sherk A, Stockwell T, Chikritzhs T, et al. Alcohol Consumption and the Physical Availability of Take-Away Alcohol: Systematic Reviews and Meta-Analyses of the Days and Hours of Sale and Outlet Density. J Stud Alcohol Drugs. Jan 2018;79(1):58-67.

Primary studies, all (37)

1. Ahern J, Margerison-Zilko C, Hubbard A, Galea S. Alcohol outlets and binge drinking in urban neighborhoods: the implications of nonlinearity for intervention and policy. American journal of public health. Apr 2013;103(4):e81-7. doi:10.2105/AJPH.2012.301203
2. Blake D, Nied A. The demand for alcohol in the United Kingdom. Applied Economics. 1997;29(12):1655-1672. doi:10.1080/00036849700000041

3. Blose JO, Holder HD. Public availability of distilled spirits: structural and reported consumption changes associated with liquor-by-the-drink. J Stud Alcohol. Jul 1987;48(4):371-9. doi:10.15288/jsa.1987.48.371
4. Brenner AB, Borrell LN, Barrientos-Gutierrez T, Diez Roux AV. Longitudinal associations of neighborhood socioeconomic characteristics and alcohol availability on drinking: Results from the Multi-Ethnic Study of Atherosclerosis (MESA). Soc Sci Med. Nov 2015;145:17-25. doi:10.1016/j.socscimed.2015.09.030
5. Chen M-J, Grube JW, Gruenewald PJ. Community alcohol outlet density and underage drinking. Addiction. Feb 2010;105(2):270-8. doi:10.1111/j.1360-0443.2009.02772.x
6. Connor JL, Kypri K, Bell ML, Cousins K. Alcohol outlet density, levels of drinking and alcohol-related harm in New Zealand: a national study. Journal of epidemiology and community health. Oct 2011;65(10):841-6. doi:10.1136/jech.2009.104935
7. Cooper HL, Bonney LE, Ross Z, et al. The aftermath of public housing relocation: relationship to substance misuse. Drug Alcohol Depend. Nov 1 2013;133(1):37-44. doi:10.1016/j.drugalcdep.2013.06.003
8. Gruenewald PJ, Johnson FW, Treno AJ. Outlets, drinking and driving: a multilevel analysis of availability. J Stud Alcohol. Jul 2002;63(4):460-8. doi:10.15288/jsa.2002.63.460
9. Gruenewald PJ, Ponicki WR, Holder HD. The relationship of outlet densities to alcohol consumption: a time series cross-sectional analysis. Alcoholism, clinical and experimental research. Feb 1993;17(1):38-47. doi:10.1111/j.1530-0277.1993.tb00723.x
10. Gruenewald PJ, Remer LG, LaScala EA. Testing a social ecological model of alcohol use: the California 50-city study. Addiction. May 2014;109(5):736-45. doi:10.1111/add.12438
11. Halonen JI, Kivimaki M, Pentti J, et al. Association of the availability of beer, wine, and liquor outlets with beverage-specific alcohol consumption: a cohort study. Alcoholism, clinical and experimental research. Apr 2014;38(4):1086-93. doi:10.1111/acer.12350
12. Hoadley JF, Fuchs BC, Holder HD. The Effect of Alcohol Beverage Restrictions on Consumption: A 25-Year Longitudinal Analysis. The American Journal of Drug and Alcohol Abuse. 1984;10(3):375-401. doi:10.3109/00952998409001478
13. Huckle T, Huakau J, Sweetsur P, Huisman O, Casswell S. Density of alcohol outlets and teenage drinking: living in an alcogenic environment is associated with higher consumption in a metropolitan setting. Addiction. Oct 2008;103(10):1614-21. doi:10.1111/j.1360-0443.2008.02318.x
14. Kuntsche E, Kuendig H, Gmel G. Alcohol outlet density, perceived availability and adolescent alcohol use: a multilevel structural equation model. Journal of epidemiology and community health. Sep 2008;62(9):811-6. doi:10.1136/jech.2007.065367
15. Kuntsche EN, Kuendig H. Do school surroundings matter? Alcohol outlet density, perception of adolescent drinking in public, and adolescent alcohol use. Addict Behav. Jan 2005;30(1):151-8. doi:10.1016/j.addbeh.2004.04.021
16. Kwate NOA, Meyer IH. Association between residential exposure to outdoor alcohol advertising and problem drinking among African American women in New York City. American journal of public health. Feb 2009;99(2):228-30. doi:10.2105/AJPH.2007.132217
17. Kypri K, Bell ML, Hay GC, Baxter J. Alcohol outlet density and university student drinking: a national study. Addiction. Jul 2008;103(7):1131-8. doi:10.1111/j.1360-0443.2008.02239.x
18. Livingston M, Laslett A-M, Dietze P. Individual and community correlates of young people's high-risk drinking in Victoria, Australia. Drug Alcohol Depend. Dec 1 2008;98(3):241-8. doi:10.1016/j.drugalcdep.2008.06.002

19. Mäkelä P. Whose drinking does the liberalization of alcohol policy increase? Change in alcohol consumption by the initial level in the Finnish panel survey in 1968 and 1969. Addiction. Jun 2002;97(6):701-6. doi:10.1046/j.1360-0443.2002.00144.x
20. McCornac DC, Filante RW. The demand for distilled spirits: an empirical investigation. J Stud Alcohol. 1984;45(2):176-178. doi:10.15288/jsa.1984.45.176
21. Nordlund S. Effects of Increasing Availability of Wine and Spirits in Norway. Nordic Studies on Alcohol and Drugs. 2010/04/01 2010;27(2):127-140. doi:10.1177/145507251002700205
22. Picone G, MacDougald J, Sloan F, Platt A, Kertesz S. The effects of residential proximity to bars on alcohol consumption. Int J Health Care Finance Econ. Dec 2010;10(4):347-67. doi:10.1007/s10754-010-9084-0
23. Pollack CE, Cubbin C, Ahn D, Winkleby M. Neighbourhood deprivation and alcohol consumption: does the availability of alcohol play a role? Int J Epidemiol. Aug 2005;34(4):772-80. doi:10.1093/ije/dyi026
24. Rootman I, Oakey J. School and Community Correlates of Alcohol Use and Abuse Among Alberta Junior High School Students. Canadian Journal of Public Health / Revue Canadienne de Sante'e Publique. 1973;64(4):351-359.
25. Schonlau M, Scribner R, Farley TA, et al. Alcohol outlet density and alcohol consumption in Los Angeles county and southern Louisiana. Geospat Health. Nov 2008;3(1):91-101. doi:10.4081/gh.2008.235
26. Scribner R, Mason K, Theall K, et al. The contextual role of alcohol outlet density in college drinking. J Stud Alcohol Drugs. Jan 2008;69(1):112-20. doi:10.15288/jsad.2008.69.112
27. Scribner RA, Cohen DA, Fisher W. Evidence of a structural effect for alcohol outlet density: a multilevel analysis. Alcoholism, clinical and experimental research. Feb 2000;24(2):188-95. doi:10.1111/j.1530-0277.2000.tb04590.x
28. Shimotsu ST, Jones-Webb RJ, MacLehose RF, Nelson TF, Forster JL, Lytle LA. Neighborhood socioeconomic characteristics, the retail environment, and alcohol consumption: a multilevel analysis. Drug Alcohol Depend. Oct 1 2013;132(3):449-56. doi:10.1016/j.drugalcdep.2013.03.010
29. Stockwell T, Zhao J, Macdonald S, Pakula B, Gruenewald P, Holder H. Changes in per capita alcohol sales during the partial privatization of British Columbia's retail alcohol monopoly 2003-2008: a multi-level local area analysis. Addiction. Nov 2009;104(11):1827-36. doi:10.1111/j.1360-0443.2009.02658.x
30. Theall KP, Lancaster BP, Lynch S, et al. The neighborhood alcohol environment and at-risk drinking among African-Americans. Alcoholism, clinical and experimental research. May 2011;35(5):996-1003. doi:10.1111/j.1530-0277.2010.01430.x
31. Tobler AL, Komro KA, Maldonado-Molina MM. Relationship between neighborhood context, family management practices and alcohol use among urban, multi-ethnic, young adolescents. Prev Sci. Dec 2009;10(4):313-24. doi:10.1007/s11121-009-0133-1
32. Trolldal B. Availability and Sales of Alcohol in Four Canadian Provinces: A Time-Series Analysis. Contemporary Drug Problems. 2005/09/01 2005;32(3):343-372. doi:10.1177/009145090503200302
33. Truong KD, Sturm R. Alcohol environments and disparities in exposure associated with adolescent drinking in California. American journal of public health. Feb 2009;99(2):264-70. doi:10.2105/AJPH.2007.122077
34. van Oers JA, Garretsen HF. The geographic relationship between alcohol use, bars, liquor shops and traffic injuries in Rotterdam. J Stud Alcohol. Nov 1993;54(6):739-44. doi:10.15288/jsa.1993.54.739
35. Wagenaar AC, Langley JD. Alcohol licensing system changes and alcohol consumption: introduction of wine into New Zealand grocery stores. Addiction. Jun 1995;90(6):773-83. doi:10.1046/j.1360-0443.1995.9067734.x

36. Weitzman ER, Nelson TF, Wechsler H. Taking up binge drinking in college: the influences of person, social group, and environment. J Adolesc Health. Jan 2003;32(1):26-35. doi:10.1016/s1054-139x(02)00457-3

37. Xie X, Mann RE, Smart RG. The direct and indirect relationships between alcohol prevention measures and alcoholic liver cirrhosis mortality. J Stud Alcohol. Jul 2000;61(4):499-506. doi:10.15288/jsa.2000.61.499

Addressing alcohol availability: retail privatization/monopolization

Umbrella reviews, included (2)

Martineau, Tyner, 2013
- Number of reviews: 2 (Hahn, Middleton et al., 2012; Campbell, Hahn, et al., 2009)

Anderson, Chisholm, Fuhr, 2009
- Number of reviews: 0
- Number of primary studies: 1 (Holder, Agardh, 2008)

Reviews, included (1)

Hahn, Middleton et al., 2012
- Number of primary studies: 15 (Fitzgerald & Mulford, 1992, 1993a, 1993b; Holder & Wagenaar, 1990; Macdonald, 1986; Makela, 2002; Mulford & Fitzgerald, 1988; Mulford et al., 1992; Popova et al. 2012; Smart, 1986; Trolldal, 2005a, 2005b; Wagenaar & Holder, 1991, 1995)

Umbrella reviews, all (2)

1. Anderson P, Chisholm D, Fuhr DC. Effectiveness and cost-effectiveness of policies and programmes to reduce the harm caused by alcohol. The Lancet. 2009;373(9682):2234-2246. doi:10.1016/S0140-6736(09)60744-3
2. Martineau F, Tyner E, Lorenc T, Petticrew M, Lock K. Population-level interventions to reduce alcohol-related harm: an overview of systematic reviews. Prev Med. Oct 2013;57(4):278-96. doi:10.1016/j.ypmed.2013.06.019

Reviews, all (1)

1. Hahn RA, Middleton JC, Elder R, et al. Effects of alcohol retail privatization on excessive alcohol consumption and related harms: a community guide systematic review. Am J Prev Med. Apr 2012;42(4):418-27. doi:10.1016/j.amepre.2012.01.002
2. Campbell CA, Hahn RA, Elder R, et al. The effectiveness of limiting alcohol outlet density as a means of reducing excessive alcohol consumption and alcohol-related harms. Am J Prev Med. Dec 2009;37(6):556-69. doi:10.1016/j.amepre.2009.09.028

Primary studies, all (15)

1. Fitzgerald JL, Mulford HA. Consequences of increasing alcohol availability: the Iowa experience revisited. British journal of addiction. Feb 1992;87(2):267-74. doi:10.1111/j.1360-0443.1992.tb02701.x
2. Fitzgerald JL, Mulford HA. Privatization, price and cross-border liquor purchases. J Stud Alcohol. Jul 1993;54(4):462-4. doi:10.15288/jsa.1993.54.462
3. Fitzgerald JL, Mulford HA. Alcohol availability, drinking contexts and drinking problems: the Iowa experience. J Stud Alcohol. May 1993;54(3):320-5. doi:10.15288/jsa.1993.54.320
4. Holder H, Agardh E, Högberg P, et al. Alcohol Monopoly and Public Health: Potential effects of privatization of the Swedish alcohol retail monopoly. Swedish National Institute of Public Health; 2008.
5. Holder HD, Wagenaar AC. Effects of the elimination of a state monopoly on distilled spirits' retail sales: a time-series analysis of Iowa. British journal of addiction. Dec 1990;85(12):1615-25. doi:10.1111/j.1360-0443.1990.tb01651.x
6. Macdonald S. The impact of increased availability of wine in grocery stores on consumption: four case histories. British journal of addiction. Jun 1986;81(3):381-7. doi:10.1111/j.1360-0443.1986.tb00344.x

7. Makela P. Whose drinking does the liberalization of alcohol policy increase? Change in alcohol consumption by the initial level in the Finnish panel survey in 1968 and 1969. Addiction. Jun 2002;97(6):701-6. doi:10.1046/j.1360-0443.2002.00144.x
8. Mulford HA, Fitzgerald JL. Consequences of increasing off-premise wine outlets in Iowa. British journal of addiction. Nov 1988;83(11):1271-9. doi:10.1111/j.1360-0443.1988.tb03038.x
9. Mulford HA, Ledolter J, Fitzgerald JL. Alcohol availability and consumption: Iowa sales data revisited. J Stud Alcohol. Sep 1992;53(5):487-94. doi:10.15288/jsa.1992.53.487
10. Popova S, Patra J, Sarnocinska-Hart A, Gnam WH, Giesbrecht N, Rehm J. Cost of privatisation versus government alcohol retailing systems: Canadian example. Drug Alcohol Rev. Jan 2012;31(1):4-12. doi:10.1111/j.1465-3362.2010.00276.x
11. Smart RG. The impact on consumption of selling wine in grocery stores. Alcohol Alcohol. 1986;21(3):233-6. doi:10.1093/oxfordjournals.alcalc.a044618
12. Trolldal B. An investigation of the effect of privatization of retail sales of alcohol on consumption and traffic accidents in Alberta, Canada. Addiction. May 2005;100(5):662-71. doi:10.1111/j.1360-0443.2005.01049.x
13. Trolldal B. The privatization of wine sales in Quebec in 1978 and 1983 to 1984. Alcoholism, clinical and experimental research. Mar 2005;29(3):410-6. doi:10.1097/01.alc.0000156084.27547.ec
14. Wagenaar AC, Holder HD. A change from public to private sale of wine: results from natural experiments in Iowa and West Virginia. J Stud Alcohol. Mar 1991;52(2):162-73. doi:10.15288/jsa.1991.52.162
15. Wagenaar AC, Holder HD. Changes in alcohol consumption resulting from the elimination of retail wine monopolies: results from five U.S. states. J Stud Alcohol. Sep 1995;56(5):566-72. doi:10.15288/jsa.1995.56.566

Addressing alcohol marketing: self-regulation

Umbrella reviews, included

Anderson, Chisholm, Fuhr, 2009
- Number of reviews: 0
- Number of primary studies: 1 (Jones, Hall, Munro, 2008)

Booth, Brennan et al., 2008
- Number of reviews: 0
- Number of primary studies: 0

Umbrella reviews, all (2)

1. Anderson P, Chisholm D, Fuhr DC. Effectiveness and cost-effectiveness of policies and programmes to reduce the harm caused by alcohol. The Lancet. 2009;373(9682):2234-2246. doi:10.1016/S0140-6736(09)60744-3
2. Booth A, Brennan A, Meier P, et al. Independent review of the effects of alcohol pricing and promotion: part a – systematic reviews. 2008.

Primary studies, all (1)

1. Jones SC, Hall D, Munro G. How effective is the revised regulatory code for alcohol advertising in Australia? Drug Alcohol Rev. Jan 2008;27(1):29-38. doi:10.1080/09595230701499175

Addressing alcohol marketing: advertising from government authorities to reduce alcohol use

Umbrella reviews, included (4)

Stockings, Hall et al., 2016
- Number of reviews: 1 (Martineau, Tyner et al., 2013)

Martineau, Tyner et al., 2013
- Number of reviews: 3 (Derzon, Lipsey 2001, Snyder et al. 2004, Stead et al. 2007)

Anderson, Chisholm, Fuhr, 2009
- Number of reviews: 1 (Babor et al., 2003)

Booth, Brennan et al., 2008
- Number of reviews: 0
- Number of primary studies: 7 (Adnsager, Austin, Pinkleton 2001, Creyer, Kozup, Burton 2002, Flynn et al. 2006, Garretson, Burton 1998, Pinkleton et al., 2001, Russell, Russell 2008, Thomsen, Fulton 2007)

Reviews, included (2)

Young, Lewis et al., 2018
- Number of primary studies: 13 (Barber, Bradshaw, Walsh 1989, Barber, Grichting 1990, Dixon et al. 2015, Flynn et al. 2006, Gronbaek et al. 2001, Hanson, Winberg, Elliott 2012, Karlsson et al. 2005, Kypri et al. 2005, Plant, Pirie and Kreitman 1979, Scheier, Grenard 2010, Trees 2015, van Leeuwen, Renes, Leeuwis 2013, Wallack, Barrows 1982)

Bryden, Roberts et al., 2012
- Number of primary studies: 2 (Flynn et al. 2006, Kuo et al. 2003)

Umbrella reviews, all (4)

1. Anderson P, Chisholm D, Fuhr DC. Effectiveness and cost-effectiveness of policies and programmes to reduce the harm caused by alcohol. The Lancet. 2009;373(9682):2234-2246. doi:10.1016/S0140-6736(09)60744-3
2. Booth A, Brennan A, Meier P, et al. Independent review of the effects of alcohol pricing and promotion: part a – systematic reviews. 2008.
3. Martineau F, Tyner E, Lorenc T, Petticrew M, Lock K. Population-level interventions to reduce alcohol-related harm: an overview of systematic reviews. Prev Med. Oct 2013;57(4):278-96. doi:10.1016/j.ypmed.2013.06.019
4. Stockings E, Hall WD, Lynskey M, et al. Prevention, early intervention, harm reduction, and treatment of substance use in young people. Lancet Psychiatry. Mar 2016;3(3):280-96. doi:10.1016/s2215-0366(16)00002-x

Reviews, all (5)

1. Bryden A, Roberts B, McKee M, Petticrew M. A systematic review of the influence on alcohol use of community level availability and marketing of alcohol. Health Place. Mar 2012;18(2):349-57. doi:10.1016/j.healthplace.2011.11.003
2. Derzon JH, Lipsey MW. Chapter 11: A Meta-analysis of the Effectiveness of Mass-Communication for Changing Substance-use Knowledge, Attitudes, and Behavior. In: Crano WD, Burgoon M, Oskamp S, eds. Mass Media and Drug Prevention: Classic and Contemporary Theories and Research. 1st ed. Psychology Press; 2001.
3. Snyder LB, Hamilton MA, Mitchell EW, Kiwanuka-Tondo J, Fleming-Milici F, Proctor D. A meta-analysis of the effect of mediated health communication campaigns on behavior change in the United States. J Health Commun. 2004;9 Suppl 1:71-96. doi:10.1080/10810730490271548

4. Stead M, Gordon R, Angus K, McDermott L. A systematic review of social marketing effectiveness. Health Education. 2007;107(2):126-191. doi:10.1108/09654280710731548

5. Young B, Lewis S, Katikireddi SV, et al. Effectiveness of Mass Media Campaigns to Reduce Alcohol Consumption and Harm: A Systematic Review. Alcohol Alcohol. May 1 2018;53(3):302-316. doi:10.1093/alcalc/agx094

Primary studies, all (20)

1. Adnsager JL, Austin EW, Pinkleton BE. Questioning the Value of Realism: Young Adults' Processing of Messages in Alcohol-Related Public Service Announcements and Advertising. Journal of Communication. 2001;51(1):121-142. doi:10.1111/j.1460-2466.2001.tb02875.x
2. Barber JG, Bradshaw R, Walsh C. Reducing alcohol consumption through television advertising. J Consult Clin Psychol. Oct 1989;57(5):613-8. doi:10.1037//0022-006x.57.5.613
3. Barber JJ, Grichting WL. Australia's media campaign against drug abuse. The International journal of the addictions. Jun 1990;25(6):693-708. doi:10.3109/10826089009061328
4. Creyer EH, Kozup JC, Burton S. An Experimental Assessment of the Effects of Two Alcoholic Beverage Health Warnings Across Countries and Binge-Drinking Status. Journal of Consumer Affairs. 2002/12/01 2002;36(2):171-202. doi:10.1111/j.1745-6606.2002.tb00429.x
5. Dixon HG, Pratt IS, Scully ML, et al. Using a mass media campaign to raise women's awareness of the link between alcohol and cancer: cross-sectional pre-intervention and post-intervention evaluation surveys. BMJ Open. Mar 11 2015;5(3):e006511. doi:10.1136/bmjopen-2014-006511
6. Flynn BS, Worden JK, Bunn JY, Dorwaldt AL, Dana GS, Callas PW. Mass media and community interventions to reduce alcohol use by early adolescents. Journal of Studies on Alcohol. 2006/01/01 2006;67(1):66-74. doi:10.15288/jsa.2006.67.66
7. Garretson JA, Burton S. Alcoholic Beverage Sales Promotion: An Initial Investigation of the Role of Warning Messages and Brand Characters among Consumers over and under the Legal Drinking Age. Journal of Public Policy & Marketing. 1998/03/01 1998;17(1):35-47. doi:10.1177/074391569801700105
8. Gronbaek M, Stroger U, Strunge H, Moller L, Graff V, Iversen L. Impact of a 10-year nation-wide alcohol campaign on knowledge of sensible drinking limits in Denmark. Eur J Epidemiol. 2001;17(5):423-7. doi:10.1023/a:1013765827585
9. Hanson JD, Winberg A, Elliott A. Development of a media campaign on fetal alcohol spectrum disorders for Northern Plains American Indian communities. Health Promot Pract. Nov 2012;13(6):842-7. doi:10.1177/1524839911404232
10. Karlsson T, Raitasalo K, Holmila M, Koski-Jannes A, Ollikainen H, Simpura J. The impact of a self-help pamphlet on reducing risk drinking among 30- to 49-year-old men in Helsinki, Finland. Subst Use Misuse. 2005;40(12):1831-47. doi:10.1080/10826080500318533
11. Kuo M, Wechsler H, Greenberg P, Lee H. The marketing of alcohol to college students: the role of low prices and special promotions. Am J Prev Med. Oct 2003;25(3):204-11. doi:10.1016/s0749-3797(03)00200-9
12. Kypri K, Dean J, Kirby S, Harris J, Kake T. 'Think before you buy under-18s drink': evaluation of a community alcohol intervention. Drug Alcohol Rev. Jan 2005;24(1):13-20. doi:10.1080/09595230500102731
13. Pinkleton BE, Weintraub Austin E, Fujioka Y. The Relationship of Perceived Beer Ad and PSA Quality to High School Students' Alcohol-Related Beliefs and Behaviors. Journal of Broadcasting & Electronic Media. 2001/12/01 2001;45(4):575-597. doi:10.1207/s15506878jobem4504_3
14. Plant MA, Pirie F, Kreitman N. Evaluation of the Scottish Health Education Unit's 1976 campaign on alcoholism. Social psychiatry. 1979/03/01 1979;14(1):11-24. doi:10.1007/BF00583569

15. Russell DW, Russell CA. Embedded Alcohol Messages in Television Series: The Interactive Effect of Warnings and Audience Connectedness on Viewers' Alcohol Beliefs. Journal of Studies on Alcohol and Drugs. 2008/05/01 2008;69(3):459-467. doi:10.15288/jsad.2008.69.459
16. Scheier LM, Grenard JL. Influence of a nationwide social marketing campaign on adolescent drug use. J Health Commun. Apr 2010;15(3):240-71. doi:10.1080/10810731003686580
17. Thomsen SR, Fulton K. Adolescents' Attention to Responsibility Messages in Magazine Alcohol Advertisements: An Eye-Tracking Approach. Journal of Adolescent Health. 2007/07/01/ 2007;41(1):27-34. doi:10.1016/j.jadohealth.2007.02.014
18. Trees K. Mobile media: communicating with and by Indigenous youth about alcohol. Australian Aboriginal Studies. 2015;2015:97+.
19. van Leeuwen L, Renes RJ, Leeuwis C. Televised entertainment-education to prevent adolescent alcohol use: perceived realism, enjoyment, and impact. Health Educ Behav. Apr 2013;40(2):193-205. doi:10.1177/1090198112445906
20. Wallack L, Barrows DC. Evaluating primary prevention: the california "winners" alcohol program. Int Q Community Health Educ. Jan 1 1982;3(4):307-36. doi:10.2190/YJDA-24KY-TTUC-9TRA

Addressing alcohol marketing: regulating the volume of advertising from alcohol manufacturers

Umbrella reviews, included (9)

Siegfried, Parry, 2019
- Number of reviews: 1 (Siegfried et al., 2014)

Burton, Henn et al., 2017
- Number of reviews: 2 (Organisation for Economic Co-operation and Development 2015, Siegfried et al. 2014)
- Number of primary studies: 3 (Cobiac et al. 2009, Holm et al. 2014, Meier et al. 2009)

Petticrew, Shemilt et al., 2017
- Number of reviews: 1 (Siegfried et al., 2014)

Stockings, Hall et al., 2016
- Number of reviews: 1 (Siegfried et al., 2014; Anderson et al., 2009)

Knai, Petticrew et al., 2015
- Number of reviews: 0

Martineau, Tyner et al., 2013
- Number of reviews: 1 (Booth et al., 2008)

Jackson, Johnson et al., 2010
- Number of reviews: 2 (Anderson et al., 2009; Booth et al., 2008)

Anderson, Chisholm, Fuhr, 2009
- Number of reviews: 0

Booth, Brennan et al., 2008
- Number of reviews: 0
- Number of primary studies: 4 (Nelson, Young 2001, Nelson 2003, Saffer 1991, Saffer, Dave 2002)

Reviews, included (5)

Manthey, Jacobsen et al., 2024
- Number of primary studies: 11 (Assanangkornchai et al., 2020; Kypri et al., 2018; Leal-Lopez et al., 2020; Makowsky, Whitehead 1991; Matrai et al., 2014; Nelson, 2003; Nelson, 2010; Ogborne, Smart 1980; Rossow, 2021; Saffer, Dave, 2002; Smart, Cutler, 1976)

Esser, Jernigan, 2018
- Number of reviews: 1 (Anderson et al, 2009)
- Number of primary studies: 3 (Chisholm et al. 2004, Hollingworth et al. 2006, Holm et al. 2014)

Siegfried, Pienaar et al., 2014
- Number of primary studies: 3 (Makowsky, Whitehead 1991, Ogborne, Smart 1980, Smart, Cutler 1976)

Hastings, Anderson et al., 2005
- Number of primary studies: 3 (Nelson 2003, Saffer 1991, Saffer, Dave 2002)

Grube, Waiters, 2005
- Number of primary studies: 6 (Makowsky, Whitehead 1991, Nelson, Young 2001, Ornstein, Hanssens 1985, Saffer 1991, Saffer, Dave 2002, Young 1993)

Umbrella reviews, all (9)

1. Anderson P, Chisholm D, Fuhr DC. Effectiveness and cost-effectiveness of policies and programmes to reduce the harm caused by alcohol. The Lancet. 2009;373(9682):2234-2246. doi:10.1016/S0140-6736(09)60744-3

2. Booth A, Brennan A, Meier P, et al. Independent review of the effects of alcohol pricing and promotion: part a – systematic reviews. 2008.
3. Burton R, Henn C, Lavoie D, et al. A rapid evidence review of the effectiveness and cost-effectiveness of alcohol control policies: an English perspective. Lancet. Apr 15 2017;389(10078):1558-1580. doi:10.1016/S0140-6736(16)32420-5
4. Jackson R, Johnson M, Campbell F, et al. Interventions on Control of Alcohol Price, Promotion and Availability for Prevention of Alcohol Use Disorders in Adults and Young People. 2010.
5. Knai C, Petticrew M, Durand MA, Eastmure E, Mays N. Are the Public Health Responsibility Deal alcohol pledges likely to improve public health? An evidence synthesis. Addiction. Aug 2015;110(8):1232-46. doi:10.1111/add.12855
6. Martineau F, Tyner E, Lorenc T, Petticrew M, Lock K. Population-level interventions to reduce alcohol-related harm: an overview of systematic reviews. Prev Med. Oct 2013;57(4):278-96. doi:10.1016/j.ypmed.2013.06.019
7. Petticrew M, Shemilt I, Lorenc T, et al. Alcohol advertising and public health: systems perspectives versus narrow perspectives. Journal of epidemiology and community health. Mar 2017;71(3):308-312. doi:10.1136/jech-2016-207644
8. Siegfried N, Parry C. Do alcohol control policies work? An umbrella review and quality assessment of systematic reviews of alcohol control interventions (2006 - 2017). PLoS One. 2019;14(4):e0214865. doi:10.1371/journal.pone.0214865
9. Stockings E, Hall WD, Lynskey M, et al. Prevention, early intervention, harm reduction, and treatment of substance use in young people. Lancet Psychiatry. Mar 2016;3(3):280-96. doi:10.1016/s2215-0366(16)00002-x

Reviews, all (6)

1. Esser MB, Jernigan DH. Policy Approaches for Regulating Alcohol Marketing in a Global Context: A Public Health Perspective. Annu Rev Public Health. Apr 1 2018;39:385-401. doi:10.1146/annurev-publhealth-040617-014711
2. Grube JW, Waiters E. Alcohol in the media: content and effects on drinking beliefs and behaviors among youth. Adolesc Med Clin. Jun 2005;16(2):327-43, viii. doi:10.1016/j.admecli.2005.02.005
3. Hastings G, Anderson S, Cooke E, Gordon R. Alcohol marketing and young people's drinking: a review of the research. J Public Health Policy. Sep 2005;26(3):296-311. doi:10.1057/palgrave.jphp.3200039
4. Manthey J, Jacobsen B, Klinger S, Schulte B, Rehm J. Restricting alcohol marketing to reduce alcohol consumption: A systematic review of the empirical evidence for one of the 'best buys'. Addiction. 2024;119(5):799-811. doi:10.1111/add.16411
5. Organisation for Economic Co-operation and Development. Tackling Harmful Alcohol Use: Economics and Public Health Policy. 2015.
6. Siegfried N, Pienaar DC, Ataguba JE, et al. Restricting or banning alcohol advertising to reduce alcohol consumption in adults and adolescents. Cochrane Database Syst Rev. Nov 4 2014;(11):CD010704. doi:10.1002/14651858.CD010704.pub2

Primary studies, all (20)

1. Assanangkornchai S, Saingam D, Jitpiboon W, Geater AF. Comparison of drinking prevalence among Thai youth before and after implementation of the Alcoholic Beverage Control Act. Am J Drug Alcohol Abuse. May 3 2020;46(3):325-332. doi:10.1080/00952990.2019.1692213

2. Chisholm D, Rehm J, Van Ommeren M, Monteiro M. Reducing the global burden of hazardous alcohol use: a comparative cost-effectiveness analysis. J Stud Alcohol. Nov 2004;65(6):782-93. doi:10.15288/jsa.2004.65.782
3. Cobiac L, Vos T, Doran C, Wallace A. Cost-effectiveness of interventions to prevent alcohol-related disease and injury in Australia. Addiction. Oct 2009;104(10):1646-55. doi:10.1111/j.1360-0443.2009.02708.x
4. Hollingworth W, Ebel BE, McCarty CA, Garrison MM, Christakis DA, Rivara FP. Prevention of deaths from harmful drinking in the United States: the potential effects of tax increases and advertising bans on young drinkers. J Stud Alcohol. Mar 2006;67(2):300-8. doi:10.15288/jsa.2006.67.300
5. Holm AL, Veerman L, Cobiac L, Ekholm O, Diderichsen F. Cost-effectiveness of preventive interventions to reduce alcohol consumption in Denmark. PLoS One. 2014;9(2):e88041. doi:10.1371/journal.pone.0088041
6. Kypri K, Maclennan B, Cousins K, Connor J. Hazardous Drinking among Students over a Decade of University Policy Change: Controlled Before-and-After Evaluation. Int J Environ Res Public Health. Sep 28 2018;15(10)doi:10.3390/ijerph15102137
7. Leal-Lopez E, Moreno-Maldonado C, Inchley J, et al. Association of alcohol control policies with adolescent alcohol consumption and with social inequality in adolescent alcohol consumption: A multilevel study in 33 countries and regions. The International journal on drug policy. Oct 2020;84:102854. doi:10.1016/j.drugpo.2020.102854
8. Makowsky CR, Whitehead PC. Advertising and alcohol sales: a legal impact study. J Stud Alcohol. Nov 1991;52(6):555-67. doi:10.15288/jsa.1991.52.555
9. Matrai S, Casajuana C, Allamani A, et al. The relationships between the impact of alcoholic beverage control policies, selected contextual determinants, and alcohol drinking in Spain. Subst Use Misuse. Oct 2014;49(12):1665-83. doi:10.3109/10826084.2014.913398
10. Meier P, Brennan A, Purshouse R, Taylor K, Rafia R. Independent Review of the Effects of Alcohol Pricing and Promotion: Part B. Modelling the Potential Impact of Pricing and Promotion Policies for Alcohol in England: Results from the Sheffield Alcohol Policy Model Version 2008(1-1). 2009.
11. Nelson JP. Advertising Bans, Monopoly, and Alcohol Demand: Testing for Substitution Effects using State Panel Data. Review of Industrial Organization. 2003;22(1):1-25. doi:10.1023/A:1022184014407
12. Nelson JP. Alcohol advertising bans, consumption and control policies in seventeen OECD countries, 1975–2000. Applied Economics. 2010/03/01 2010;42(7):803-823. doi:10.1080/00036840701720952
13. Nelson JP, Young DJ. Do advertising bans work? An international comparison. International Journal of Advertising. 2001/01/01 2001;20(3):273-296. doi:10.1080/02650487.2001.11104894
14. Ogborne AC, Smart RG. Will restrictions on alcohol advertising reduce alcohol consumption? British journal of addiction. Sep 1980;75(3):293-6. doi:10.1111/j.1360-0443.1980.tb01382.x
15. Ornstein SI, Hanssens DM. Alcohol Control Laws and the Consumption of Distilled Spirits and Beer. Journal of Consumer Research. 1985;12(2):200-213. doi:10.1086/208509
16. Rossow I. The alcohol advertising ban in Norway: Effects on recorded alcohol sales. Drug Alcohol Rev. Nov 2021;40(7):1392-1395. doi:10.1111/dar.13289
17. Saffer H. Alcohol advertising bans and alcohol abuse: An international perspective. Journal of health economics. 1991/05/01/ 1991;10(1):65-79. doi:10.1016/0167-6296(91)90017-H
18. Saffer H, Dave D. Alcohol consumption and alcohol advertising bans. Applied Economics. 2002;34(11):1325-1334. doi:10.1080/00036840110102743
19. Smart RG, Cutler RE. The alcohol advertising ban in British Columbia: problems and effects on beverage consumption. Br J Addict Alcohol Other Drugs. Mar 1976;71(1):13-21. doi:10.1111/j.1360-0443.1976.tb00055.x

20. Young DJ. Alcohol advertising bans and alcohol abuse: Comment. Journal of health economics. 1993/07/01/ 1993;12(2):213-228. doi:10.1016/0167-6296(93)90032-A

Addressing alcohol marketing: warning labels

Umbrella reviews, included (5)

Siegfried, Parry, 2019
- Number of reviews: 1 (Scholes-Balog, Heerde, Hemphill, 2012)

Burton, Henn et al., 2017
- Number of reviews: 2 (Scholes-Balog, Heerde, Hemphill, 2012; Stockwell, 2006)

Knai, Petticrew et al., 2015
- Number of reviews: 4 (Babor et al. 2010, International Center for Alcohol Policies 2009, Scholes-Balog, Heerde, Hemphill 2012, Wilkinson et al. 2009)

Anderson, Chisholm, Fuhr, 2009
- Number of reviews: 1 (Wilkinson, Room, 2009)

Stockwell, 2006
- Number of reviews: 6 (Agostinelli, Grube 2002, Andrews 1995, Babor et al. 2003, International Center for Alcohol Policies, 1997, Loxley et al. 2004, Stockley 2001)
- Number of primary studies: 6 (Greenfield, Graves and Kaskutas 1999, Hankin et al. 1993, Kaskutas, Greenfield, 1992, Kaskutas and Graves 1994, Kaskutas et al. 1998, MacKinnon et al. 2000)

Reviews, included (6)

Joyce, Davidson et al., 2023
- Number of primary studies: 6 (Hankin et al. 1996, Hankin et al. 1993, Hankin, Sloan, Sokol 1998, MacKinnon et al. 2001, Mayer, Smith, Scammon 1991, Zhao et al. 2020)

Clarke, Pechey et al., 2020
- Number of primary studies: 0

Hassan, Shiu, 2018
- Number of primary studies: 2 (MacKinnon et al. 2000, MacKinnon et al. 2001)

Scholes-Balog, Heerde, Hemphill, 2012
- Number of primary studies: 2 (MacKinnon et al. 2000, MacKinnon et al. 2001)

Wilkinson, Allsop et al., 2009
- Number of primary studies: 4 (Greenfield, Graves, Kaskutas 1999, Hankin et al. 1993, Kaskutas, Graves 1994, MacKinnon et al. 2001)

Wilkinson, Room, 2009
- Number of primary studies: 3 (Greenfield, Graves, Kaskutas 1999, Hankin et al. 1993, MacKinnon et al. 2000)

Umbrella reviews, all (5)

1. Anderson P, Chisholm D, Fuhr DC. Effectiveness and cost-effectiveness of policies and programmes to reduce the harm caused by alcohol. The Lancet. 2009;373(9682):2234-2246. doi:10.1016/S0140-6736(09)60744-3
2. Burton R, Henn C, Lavoie D, et al. A rapid evidence review of the effectiveness and cost-effectiveness of alcohol control policies: an English perspective. Lancet. Apr 15 2017;389(10078):1558-1580. doi:10.1016/S0140-6736(16)32420-5
3. Knai C, Petticrew M, Durand MA, Eastmure E, Mays N. Are the Public Health Responsibility Deal alcohol pledges likely to improve public health? An evidence synthesis. Addiction. Aug 2015;110(8):1232-46. doi:10.1111/add.12855

4. Siegfried N, Parry C. Do alcohol control policies work? An umbrella review and quality assessment of systematic reviews of alcohol control interventions (2006 - 2017). PLoS One. 2019;14(4):e0214865. doi:10.1371/journal.pone.0214865
5. Stockwell T. A Review of Research into the Impacts of Alcohol Warning Labels on Attitudes and Behaviour. 2006.

Reviews, all (14)

1. Agostinelli G, Grube JW. Alcohol counter-advertising and the media. A review of recent research. Alcohol Res Health. 2002;26(1):15-21.
2. Andrews JC. The Effectiveness of Alcohol Warning Lables: A Review and Extension. American Behavioral Scientist. 1995/02/01 1995;38(4):622-632. doi:10.1177/0002764295038004011
3. Babor TF, Caetano R, Casswell S, et al. Alcohol: No Ordinary Commodity: Research and Public Policy. First ed. Oxford University Press; 2003. https://academic-oup-com.libaccess.lib.mcmaster.ca/book/5789
4. Babor TF, Caetano R, Casswell S, et al. Alcohol: No Ordinary Commodity: Research and Public Policy. Second ed. Oxford University Press; 2010. https://academic-oup-com.libaccess.lib.mcmaster.ca/book/5789
5. Clarke N, Pechey E, Kosite D, et al. Impact of health warning labels on selection and consumption of food and alcohol products: systematic review with meta-analysis. Health Psychol Rev. Jul 2 2020:1-24. doi:10.1080/17437199.2020.1780147
6. Hassan LM, Shiu E. A systematic review of the efficacy of alcohol warning labels. Journal of Social Marketing. 2018;8(3):333-352. doi:10.1108/JSOCM-03-2017-0020
7. International Center for Alcohol Policies. Health Warning Labels. ICAP Reports No. 3. 1997.
8. International Center for Alcohol Policies. Health Warning Labels. 2009. https://www.webcitation.org/6WEfjZ3Nc
9. Joyce KM, Davidson M, Manly E, Stewart SH, Al-Hamdani M. A systematic review on the impact of alcohol warning labels. J Addict Dis. May 22 2023:1-24. doi:10.1080/10550887.2023.2210020
10. Loxley W, oumbourou JWT, Stockwell T, et al. The Prevention of Substance Use, Risk and Harm in Australia: a review of the evidence. 2004. https://espace.curtin.edu.au/bitstream/handle/20.500.11937/30403/19135_19135.pdf?sequence=2&isAllowed=y
11. Scholes-Balog KE, Heerde JA, Hemphill SA. Alcohol warning labels: unlikely to affect alcohol-related beliefs and behaviours in adolescents. Aust N Z J Public Health. Dec 2012;36(6):524-9. doi:10.1111/j.1753-6405.2012.00934.x
12. Stockley CS. The effectiveness of strategies such as health warning labels to reduce alcohol-related harms — an Australian perspective. International Journal of Drug Policy. 2001/07/01/2001;12(2):153-166. doi:10.1016/S0955-3959(01)00077-9
13. Wilkinson C, Allsop S, Cail D, et al. Report 1 Alcohol Warning Labels: Evidence of effectiveness on risky alcohol consumption and short term outcomes. Prepared by the National Drug Research Institute (Curtin University of Technology), in collaboration with Drug and Alcohol Office (WA), National Drug and Alcohol Research Centre (University of New South Wales), Public Health Advocacy Institute (Curtin University of Technology). Perth: National Drug Research Institute;2009.
14. Wilkinson C, Room R. Warnings on alcohol containers and advertisements: international experience and evidence on effects. Drug Alcohol Rev. 2009;28(4):426-435.

Primary studies, all (11)

1. Greenfield TK, Graves KL, Kaskutas LA. Long-term effects of alcohol warning labels: Findings from a comparison of the United States and Ontario, Canada. Psychology & Marketing. 1999/05/01 1999;16(3):261-282. doi:10.1002/(SICI)1520-6793(199905)16:3<261::AID-MAR5>3.0.CO;2-Z
2. Hankin JR, Firestone IJ, Sloan JJ, Ager JW, Sokol RJ, Martier SS. Heeding the alcoholic beverage warning label during pregnancy: multiparae versus nulliparae. Journal of Studies on Alcohol. 1996/03/01 1996;57(2):171-177. doi:10.15288/jsa.1996.57.171
3. Hankin JR, Sloan JJ, Firestone IJ, Ager JW, Sokol RJ, Martier SS. A Time Series Analysis of the Impact of the Alcohol Warning Label on Antenatal Drinking. Alcoholism: Clinical and Experimental Research. 1993/04/01 1993;17(2):284-289. doi:10.1111/j.1530-0277.1993.tb00764.x
4. Hankin JR, Sloan JJ, Sokol RJ. The Modest Impact of the Alcohol Beverage Warning Label on Drinking during Pregnancy among a Sample of African-American Women. Journal of Public Policy & Marketing. 1998/03/01 1998;17(1):61-69. doi:10.1177/074391569801700107
5. Kaskutas L, Greenfield TK. First effects of warning labels on alcoholic beverage containers. Drug and Alcohol Dependence. 1992/10/01/ 1992;31(1):1-14. doi:https://doi.org/10.1016/0376-8716(92)90002-T
6. Kaskutas LA, Graves K. Relationship between Cumulative Exposure to Health Messages and Awareness and Behavior-Related Drinking during Pregnancy. American Journal of Health Promotion. 1994/11/01 1994;9(2):115-124. doi:10.4278/0890-1171-9.2.115
7. Kaskutas LA, Greenfield T, Lee ME, Cote J. Reach and Effects of Health Messages on Drinking during Pregnancy. Journal of Health Education. 1998/02/01 1998;29(1):11-20. doi:10.1080/10556699.1998.10603292
8. MacKinnon DP, Nohre L, Cheong J, Stacy AW, Pentz MA. Longitudinal relationship between the alcohol warning label and alcohol consumption. Journal of Studies on Alcohol. 2001/03/01 2001;62(2):221-227. doi:10.15288/jsa.2001.62.221
9. MacKinnon DP, Nohre L, Pentz MA, Stacy AW. The alcohol warning and adolescents: 5-year effects. American journal of public health. Oct 2000;90(10):1589-94. doi:10.2105/ajph.90.10.1589
10. Mayer RN, Smith KR, Scammon DL. Evaluating the Impact of Alcohol Warning Labels. In: Holman RH, Solomon MR, eds. NA - Advances in Consumer Research Volume 18. Association for Consumer Research; 1991:706-714.
11. Zhao J, Stockwell T, Vallance K, Hobin E. The Effects of Alcohol Warning Labels on Population Alcohol Consumption: An Interrupted Time Series Analysis of Alcohol Sales in Yukon, Canada. Journal of Studies on Alcohol and Drugs. 2020/03/01 2020;81(2):225-237. doi:10.15288/jsad.2020.81.225
